# From vaccine to pathogen: Modeling Sabin 2 vaccine virus reversion and evolutionary epidemiology in Matlab, Bangladesh

**DOI:** 10.1093/ve/vead044

**Published:** 2023-07-08

**Authors:** Wesley Wong, Jillian Gauld, Michael Famulare

**Affiliations:** Immunology and Infectious Diseases, Harvard T.H. Chan School of Public Health, 665 Huntington Avenue, SPH 1, Boston, MA 02115, USA; Institute for Disease Modeling, Bill and Melinda Gates Foundation, 500 5th Ave N, Seattle, WA 98109, USA; Institute for Disease Modeling, Bill and Melinda Gates Foundation, 500 5th Ave N, Seattle, WA 98109, USA

**Keywords:** Sabin 2, cVDPV2, poliovirus, genetic reversion, evolution, modeling, vaccine

## Abstract

The oral poliovirus vaccines (OPVs) are one of the most effective disease eradication tools in public health. However, the OPV strains are genetically unstable and can cause outbreaks of circulating, vaccine-derived Type 2 poliovirus (cVDPV2) that are clinically indistinguishable from wild poliovirus (WPV) outbreaks. Here, we developed a Sabin 2 reversion model that simulates the reversion of Sabin 2 to reacquire a WPV-like phenotype based on the clinical differences in shedding duration and infectiousness between individuals vaccinated with Sabin 2 and those infected with WPV. Genetic reversion is informed by a canonical reversion pathway defined by three gatekeeper mutations (A481G, U2909C, and U398C) and the accumulation of deleterious nonsynonymous mutations. Our model captures essential aspects of both phenotypic and molecular evolution and simulates transmission using a multiscale transmission model that consolidates the relationships among immunity, susceptibility, and transmission risk. Despite rapid Sabin 2 attenuation reversal, we show that the emergence of a revertant virus does not guarantee a cVDPV2 outbreak. When simulating outbreaks in Matlab, Bangladesh, we found that cVDPV2 outbreaks are most likely in areas with low population-level immunity and poor sanitation. In Matlab, our model predicted that declining immunity against Type 2 poliovirus following the cessation of routine OPV vaccination was not enough to promote cVDPV2 emergence. However, cVDPV2 emergencedepended on the average viral exposure dose per contact, which was modeled as a combination of the viral concentration per fecal gram and the average fecal–oral dose per contact. These results suggest that cVDPV2 emergence risk can be mitigated by reducing the amount of infectious fecal material individuals are exposed to. Thus, a combined strategy of assessing and improving sanitation levels in conjunction with high-coverage vaccination campaigns could limit the future cVDPV2 emergence.

## Introduction

Mass immunization with oral poliovirus vaccine (OPV) has led to a >99.99 per cent drop in wild poliovirus (WPV) cases (Khan et al. 2018; Badizadegan, Kalkowska, and Thompson 2022) and the complete eradication of Type 2 and Type 3 WPVs. The OPVs are highly efficacious vaccines that confer long-lasting protective immunity (Bandyopadhyay et al. 2015). An unusual feature of these vaccines is that vaccinated individuals can shed and transmit the vaccine virus. Vaccine transmission increases population-level immunity but is problematic when viruses revert attenuation, resulting in circulating, vaccine-derived poliovirus (cVDPV). cVDPV can cause paralytic poliomyelitis cases that are clinically indistinguishable from WPV cases (Kew et al. 2005). Following certification of the eradication of Type 2 WPV, the Global Polio Eradication Initiative coordinated the global withdrawal of Sabin 2 OPV (OPV2) from routine immunization schedules in 2016 (commonly referred to as the Switch) to prevent future sources of circulating, vaccine-derived Type 2 poliovirus (cVDPV2) (World Health Organization 2013; Hampton et al. 2016; Khan et al. 2018).

Although OPV2 is no longer used in routine immunization since the Switch, cVDPV2 outbreaks have required the use of OPV2 through supplemental immunization activities (SIAs) and large-scale one-time responses. These campaigns are not without risk, and modeling before the Switch showed continued OPV2 after the Switch could seed future cVDPV2 outbreaks (Duintjer Tebbens, Hampton, and Thompson 2016a, 2016b). These findings were corroborated by a retrospective analysis of post-Switch cVDPV2 outbreaks, which concluded that most post-Switch cVDPV2 outbreaks were the result of a previous SIA (Macklin et al. 2020). Between the Switch and November 2019, 325 cases of acute flaccid paralysis associated with cVDPV2 were identified. Overall, 126 OPV2 campaigns utilizing more than 300 million doses of monovalent, Sabin 2 oral poliovirus (mOPV2) have been implemented to control cVDPV2 outbreaks linked to these cases (Macklin et al. 2020). Both OPV2 use and cVDP2 cases have dramatically increased in recent years: over 1,800 cases of cVDPV2 have been identified in 30 countries between January 2020 and April 2022, and in 2021 alone, 628 million OPV2 doses were used (Rachlin et al. 2022).

To end the cycle of OPV2 use and cVDPV2 outbreaks, a novel OPV2 vaccine (nOPV2) has been developed that is more genetically stable than OPV2 (Yeh et al. 2020). However, there is much uncertainty surrounding the properties and implementation of the nOPV2 vaccines, and previous model studies show that switching mOPV2 SIAs with nOPV2 may be insufficient to solve the current cVDPV2 problems (Kalkowska et al. 2021b). Currently, poliovirus eradication strategies still rely on OPV2 due to the limited availability of the nOPV2 vaccine. Thus, assessing the risks associated with the OPV2 remains relevant, as alternative strategies involving a mix of nOPV2 and OPV2 activities as well as the re-implementation of routine immunization of OPV2 are being considered (Kalkowska et al. 2021a).

The Sabin 2 vaccine strain was derived from a wild-type isolate (P22/P712/56) passaged in monkey kidney cells to achieve neurovirulence attenuation (Almond 1987). However, identifying the genetic mutations responsible for attenuation has been surprisingly complicated, in part because the progenitor has not been fully sequenced and because P22/P712/56 is thought to be naturally attenuated relative to other WPV strains (Sabin and Boulger 1973). Despite this, phylogenetic analyses of whole genome sequences collected from cVDPV2 outbreaks suggest a common evolutionary pathway to attenuation reversal (Stern et al. 2017). This pathway is characterized by the rapid fixation of three gatekeeper mutations, such as A481G, U2909C, and U398C, within the first few weeks after vaccination (Famulare et al. 2015; Stern et al. 2017). All three mutations have previously been implicated as molecular determinants of attenuation for Sabin 2 (Muzychenko et al. 1991; Kew et al. 2005; Duintjer Tebbens et al. 2013b; Cassemiro et al. 2016). A481G and U398C are noncoding mutations that affect the stability of a 5ʹ-hairpin structure that mediates the translation efficiency (Muzychenko et al. 1991; Ren, Moss, and Racaniello 1991; Simoes and Sarnow 1991), and U2909C is a nonsynonymous mutation in the viral protein 1 (VP1) capsid protein.

However, genetic reversion is not the sole determinant of cVDPV2 emergence (Duintjer Tebbens et al. 2013a, 2013b). Environmental sewer samples have identified revertant strains in regions with no detectable cVDPV2 cases (Asghar et al. 2014). In fact, vaccinated individuals can shed and transmit revertant viruses to other individuals (Abraham et al. 1993; Famulare et al. 2015), but whether this results in clinically detected cVDPV2 outbreaks depends on the epidemiological context. These cVDPV2 outbreaks are associated with regions with historically poor vaccination rates and low population-level immunity (Tebbens et al. 2006; Blake et al. 2018; Macklin et al. 2020). Thus, while Sabin 2 reversion is a prerequisite of cVDPV2 emergence, whether it triggers a public health crisis depends on the interactions between genetic reversion and local epidemiological conditions that include population-level immunity, demographic structure, and behavioral factors that influence how contact and transmission occur.

Mathematical models can be useful tools for assessing cVDPV2 emergence risk and characterizing the evolutionary and epidemiological factors that allow cVDPV2 outbreaks to occur. Our goal was to develop a new Sabin 2 reversion model and integrate it into our previously developed multiscale transmission model. This required first calibrating Sabin 2 molecular evolution to publicly available Sabin 2 and Sabin 2-derived sequences. Next, we re-calibrated the shedding durations and per-virion infectiousness of the individual-level infection dynamics originally reported in Famulare et al. (2018) to consider rapidly evolving viral genotypes (Materials and methods, Equations 1 and 7). Because viral shedding durations and per-virion infectiousness change over time in our model, we also needed to re-calibrate the fecal–oral dose used in the multiscale transmission model to match the longitudinal shedding profiles of household and infant cohorts immunized with OPV2 during an mOPV2 trial performed in Matlab, Bangladesh (Taniuchi et al. 2017; Famulare et al. 2018, 2021). Using this framework, we then assessed cVDPV2 emergence risk in the context of Matlab, Bangladesh, to characterize the interaction between genetics and transmission as well as to identify genetic and epidemiological conditions that could contribute to cVDPV2 emergence.

## Results

### Brief model structure overview

cVDPV2 emergence depends on both evolutionary and epidemiological factors. We previously developed an agent-based multiscale epidemiology model designed to simulate the transmission of the Sabin 2 vaccine during an mOPV2 clinical trial performed in Matlab, Bangladesh (Taniuchi et al. 2017; Famulare et al. 2018, 2021). When describing the multiscale transmission model, it is convenient to divide it into two parts: one that governs the individual infection dynamics (Famulare et al. 2018) and the other that governs the population-level immunity and contact structure (Famulare et al. 2021). At the individual level, our model simulates the interactions between immunity, shedding duration, viral shedding titer, and susceptibility based on the data collected from historical Sabin 2 clinical vaccine trials. Population-level dynamics are simulated by organizing individuals into different households and community structures.

**Figure 1.**
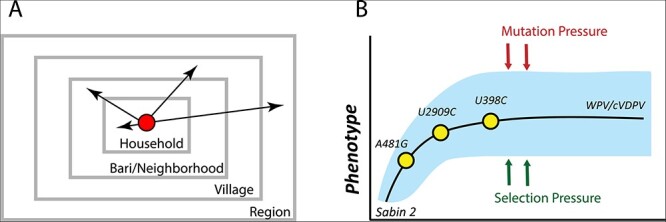
Sabin 2 transmission and reversion schematic. (A) A multiscale epidemiology model. To simulate household and community member–specific transmission rates, individuals are organized into a series of nested, demographic scales defined by households, *baris*/neighborhoods, and villages that together define the greater region. The *bari* is an intergenerational living arrangement of closely related individuals specific to Matlab, the study population the epidemiology model was calibrated to. Conceptually, it is analogous to a neighborhood. Infectious contacts occur at different rates to individuals in each of these demographic scales. During each infectious contact, infected individuals transmit a viral dose that depends on their individual viral shedding concentration and the average fecal–oral dose per contact in the population. (B) Sabin 2 reversion model. Sabin 2 evolution is modeled as a population of competing viral lineages whose average phenotypic end-state distribution is identical to that of WPV. The reversion is driven by the acquisition of three gatekeeper mutations (A481G, U2909C, and U398C) and the acquisition of deleterious mutations introduced through mutation and purged through selection.

This hierarchical, multiscale organization of individuals is then used to assign differential contact rates to different members of the populations (Fig. 1A). This multiscale structure was necessary to capture Sabin 2 vaccine virus shedding following a monovalent oral poliovirus campaign in Matlab, Bangladesh. Modeled transmission occurs through direct, fecal–oral contact, and the exposure dose is determined by the viral shedding concentration of the infected individual and a calibrated estimate of the average fecal–oral dose per contact (SI Appendix). The fecal–oral dose per contact is a model-inferred parameter that, together with the viral shedding titer, determines the total viral exposure dose per contact.

**Figure 2.**
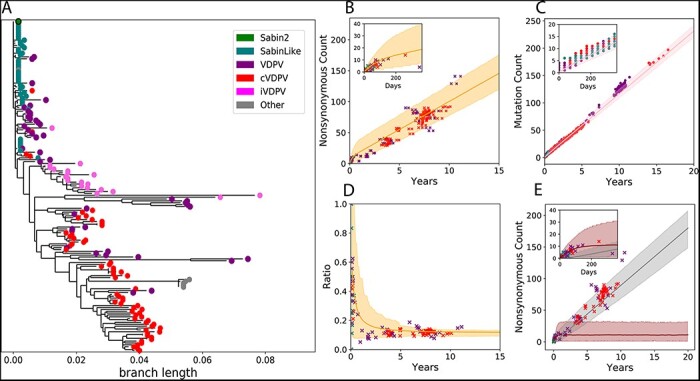
Molecular evolution. (A) A phylogenetic tree based on the nonsynonymous mutations from the 177 whole genome sequences used to calibrate our model. Samples were categorized as Sabin 2, Sabin-like, VDPV isolated from immunocompromised individuals (iVDPV), VDPV, and cVDPV2, as classified in the original publications associated with the sequences. The other category includes the Lansing and MEF-1 laboratory strains as well as several engineered strains derived from the Lansing strain. Samples classified as iVDPV or ‘other’ were excluded from the model calibration. The tree was generated by defining Sabin 2 as the root to emphasize convergent evolution across outbreaks at the amino acid level. (B) Simulated genome-wide nonsynonymous mutation accumulation over time (shading, *orange*) compared against those observed in the Sabin 2, Sabin-like, VDPV, and cVDPV2 whole genome sequences. (C) Simulated (shading, *pink*) VP1 mutation (synonymous and nonsynonymous) accumulation over time compared against the mutation counts observed in 1,643 VP1 segments. (D) The ratio of nonsynonymous and synonymous mutations throughout the genome over time. (E) Simulated genome-wide nonsynonymous counts partitioned into deleterious (dark shading, *brown*) and neutral (light shading, *gray*). Across all figures, the solid line is the mean simulation outcome, and the shaded area marks the boundaries of the middle 95 per cent. Empirical data are represented by points and colored according to the legend in A. Time was calculated by dividing the number of synonymous mutations per sample by the synonymous substitution rate (3.16E-05 substitutions/bp/day). The insets in B, C, and E are close-ups of the first year of evolution to better show short-term evolution dynamics.

Sabin 2 evolution is modeled as a population of multiple viral lineages founded by a Sabin 2 vaccine virus strain during vaccination. These viral lineages evolve independently and compete for the limited availability of naive or low-immunity individuals in the population. Phenotypic evolution is driven by the reversion of three gatekeeper mutations (A481G, U2909C, and U398C) and the accumulation of deleterious nonsynonymous mutations generated through mutation and purged through selection or genetic drift (Fig. 1B). We assume complete genetic reversion results in a viral population whose average infectiousness and shedding duration are identical to those of WPV. Due to the independent acquisition of mutations across different viruses and viral lineages, individual viruses have different infectiousness and shedding duration phenotypes even after the three gatekeeper mutations have reverted.

### Simulating Sabin 2 molecular evolution

To inform molecular evolution, intra-host substitution rates for the three gatekeeper mutations were based on estimates previously calculated from 241 VP1 segments from Sabin-like poliovirus collected during routine surveillance in Nigeria (Wassilak et al. 2011; Famulare et al. 2015). Nonsynonymous and synonymous substitution rates were calibrated (SI Appendix) to a set of whole genome sequences and VP1 capsid segments isolated from infections from confirmed Sabin 2, Sabin-like, vaccine-derived poliovirus (VDPV), and cVDPV2 cases collected from multiple countries (Materials and methods, Fig. 2). One notable feature of poliovirus evolution is the discordance between short-term evolution following vaccination and long-term evolution after prolonged circulation. Short-term evolution is dominated by the accumulation of nonsynonymous mutations, as evidenced by the increased rate of nonsynonymous mutation accumulation in the whole genome sequences during the first year of evolution (Fig. 2B), the reduction in the ratio of nonsynonymous to synonymous mutations over time (Fig. 2D), and the elevated dN/dS ratios (ratio of non-synonymous to synonymous substitutions) of Sabin-like samples compared to cVDPV2 samples (Supplementary Fig. S1).

**Figure 3.**
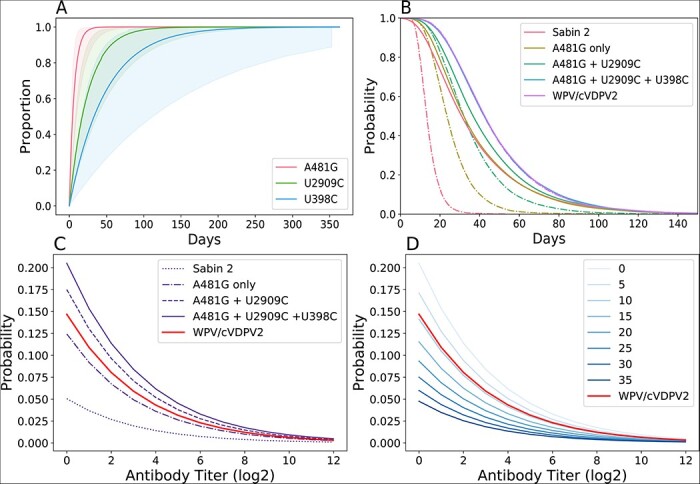
Phenotypic evolution. (A) Cumulative density reversion probability functions for the three gatekeeper mutations. The solid line is the mean and the shading is the 95 per cent confidence interval. (B) The shedding duration profile of immunologically naive individuals infected with Sabin 2, WPV/cVDPV2, and three different intermediate viral genotypes. The dotted lines are the shedding durations of the initial viral genotype, assuming no further reversion or evolution. The solid lines are the shedding durations of each viral genotype, where the reversion of new gatekeeper mutations extends the current shedding duration. Nonsynonymous mutations have no discernible phenotypic effect with regard to shedding duration. (C and D) Viral infectiousness. Each curve shows the strain-specific probability of infection, given a single CCID50 dose plotted against immunity. (C) Shows infectiousness following the acquisition of each of the three gatekeeper mutations. (D) Emphasizes the role of deleterious nonsynonymous mutations and shows the infection probability of different viral genotypes with all gatekeeper mutations and deleterious nonsynonymous mutation counts ranging from 0 to 35.

We hypothesized that the difference between short-term and long-term evolution represents a shift in the distribution of fitness effects (DOFEs) for nonsynonymous mutations. Simulated nonsynonymous mutations were divided into two categories: deleterious mutations that can cause a reduction in either infectiousness or shedding duration and neutral mutations that have no phenotypic effect. Each category is associated with a different substitution rate, and deleterious nonsynonymous mutations are purged using a recombination-like process that mimics selection and intra-species recombination within the host (SI Appendix). Deleterious nonsynonymous mutations dominate molecular evolution during the first year of evolution, while neutral nonsynonymous mutations dominate molecular evolution after prolonged circulation (Fig. 2E). Based on these simulations, we estimated that an average of nine deleterious mutations are maintained during prolonged circulation (Fig. 2E).

### Simulating Sabin 2 reversion

Gatekeeper mutation fixation is rapid and can occur within the shedding duration of a single infection (Fig. 3A). The rapid fixation of the three gatekeeper mutations presented an interesting challenge when simulating shedding duration reversion. Based on the intra-host substitution rates for each of the gatekeeper mutations (SI Appendix), 7.6 per cent of immunologically naive individuals vaccinated with Sabin 2 are expected to shed non-revertant Sabin 2, 92.4 per cent to shed virus with at least one reverted gatekeeper mutation, and 35.2 per cent to shed virus with all three gatekeeper mutations reverted (Supplementary Table S1). These results suggest that the shedding durations observed in Sabin 2 vaccine trials result from a mix of revertant and non-revertant genotypes with different shedding durations.

Genotype-to-phenotype maps for shedding duration were generated by comparing the shedding duration of naive individuals vaccinated with Sabin 2 (median 30.3 and average 36 days) with those infected with WPV (median 40.3 and average 48 days) [(Famulare et al. 2018), based on the data from Sabin 2–vaccinated individuals and WPV-exposed individuals] and estimating the change in shedding duration contributed by each gatekeeper mutation and by each deleterious nonsynonymous mutation in the genome (SI Appendix). Acquisition of the A481G mutation conferred the greatest increase in shedding duration, followed by U2909C and U398C. Interestingly, we did not identify a deleterious cost (reduction in shedding duration) associated with nonsynonymous mutation accumulation (SI Appendix).

Without evolution, our genotype-to-phenotype maps predict that immunologically naive individuals infected with unreverted Sabin 2 virus would have a median shedding duration of 13.5 days (Fig. 3B, *dashed red line*). With evolution and reversion enabled in the model, our genotype-to-phenotype maps predict that immunologically naive individuals infected with Sabin 2 virus shed for a median shedding duration of 30.3 days, which matches with that observed in vaccine trials. Those infected with a virus with all three gatekeeper mutations reverted have a shedding duration identical to WPV (median 47 days).

We next generated genotype-to-phenotype maps that describe the increase in viral infectiousness associated with Sabin 2 reversion. These genotype-to-phenotype maps were based on clinical estimates of the culture infectious dose 50% (CCID50) dose needed to establish an active infection (Fig. 3C, D). As with shedding duration, acquisition of the A481G results in the greatest increase in viral infectiousness. Unlike with shedding duration, we identified a small negative effect associated with deleterious nonsynonymous mutations resulting in a reduction in viral infectiousness (SI Appendix). As a result, the reversion of the gatekeeper mutations in the absence of any other deleterious nonsynonymous mutations results in viruses that are more infectious than the expected, average WPV phenotype (Fig. 3C). Each additional deleterious nonsynonymous mutation reduces infectiousness such that a viral genotype with all three gatekeeper mutations reverted and nine deleterious mutations is as infectious as our mean estimate for WPV (Fig. 3D).

**Figure 4.**
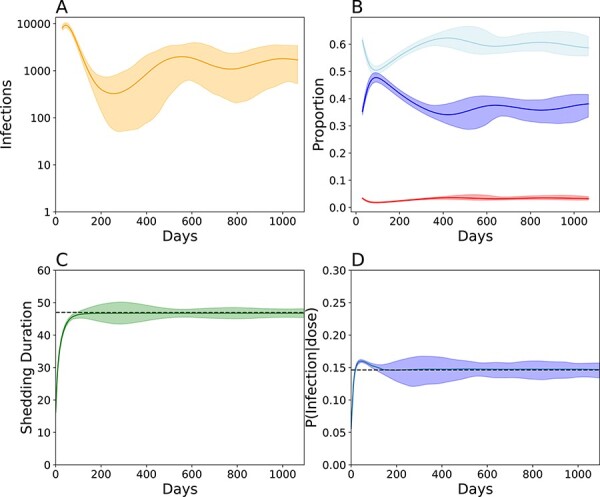
Evo-epidemiological dynamics following a mass vaccination campaign targeting 10 per cent of children under 5, five years post-vaccination cessation in Matlab, Bangladesh. Simulations assume ten times the fecal–oral dose in Matlab to ensure stable transmission and conditioned on stable, endemic transmission for at least 3 years. (A) The total number of infected individuals over time. The solid line indicates the smoothed average and the shading indicates the boundaries of the middle 95 per cent from 600 simulations. (B) Smoothed longitudinal immune profiles for individuals with low immunity (*red*, antibody titer <8), intermediate immunity (*light blue*, antibody titer >8 and <256), and high immunity (*dark blue*, antibody titer >256). (C) Smoothed shedding duration evolution in naive individuals over time. (D) Smoothed viral infectiousness evolution over time. Note the wider confidence intervals in surrounding shedding duration and shedding duration between Days 200 and 600 resulting from strong genetic drift. For B–D, the solid line denotes the simulated average and the boundaries denote the 95 per cent confidence interval around the mean. For C and D, the dashed black line indicates the average WPV phenotype.

### Examining the evolutionary epidemiology dynamics of Sabin 2 reversion

To explore how Sabin 2 reversion and transmission interact, we simulated transmission following an mOPV2 campaign targeting 10 per cent of children under 5 that occurred 1 year after vaccination cessation (Fig. 4). These simulations were performed after integrating the Sabin 2 reversion model into our previously described multiscale transmission model (Famulare et al. 2021). This required re-calibrating the fecal–oral dose to match longitudinal shedding profiles of household and infant cohorts during an mOPV2 clinical trial in Matlab, Bangladesh (Taniuchi et al. 2017) (SI Appendix). Briefly, the simulated populations consisted of 80,000 individuals spread across 45 villages of varying sizes. Here, we used a fecal–oral dose of 4.0e-6 grams per contact, ten times that of Matlab (4.0e-7 grams per contact) to ensure the sustained transmission in the simulations.

These epidemics were cyclic (Fig. 4A) and characterized by a period of rapid growth followed by a sharp decrease in infections as the availability of susceptible individuals is exhausted within the first year of transmission (Fig. 4B). Stochastic die-out occurred in 39.6 per cent of the simulations and 91.7 per cent of die-outs occurred 381.1 (294.45–536.3) days post campaign. Epidemics that avoided die-out experienced a resurgence, and 94.8 per cent of the remaining simulations established an equilibrium at around 1,300 infections. This equilibrium is maintained by the replenishment of susceptible individuals from new births and waning immunity.

When examining the phenotypic evolution of Sabin 2 in these simulations, our simulations show that complete phenotypic Sabin 2 attenuation reversion (Fig. 4C, D) occurs within the first 200 days of transmission. On average, shedding duration increases monotonically over time until the WPV phenotype is reached. The evolution of viral infectiousness was more complicated. On average, simulated viral populations were more infectious than WPV for the first 200 days of transmission. Afterwards, the average viral infectiousness decreased until it maintained an equilibrium around the expected WPV phenotype. This temporary dynamic occurs because the gatekeeper mutations fixate before the deleterious nonsynonymous mutation count reaches its equilibrium state.

However, the viral populations generated by each individual simulation were highly varied, and individual simulations could result in viral populations whose phenotypes diverged significantly from the expected, average WPV phenotype. The range of simulated phenotypic values was greatest between Days 200 and 600, where the total infection count fell below 1,000 (Supplementary Fig. S2B, C, Fig. 4C, D). This is a classic representation of genetic drift, and we calculated its impact as the variance in the average shedding duration and viral infectiousness from each simulation. As expected of genetic drift, this variance was inversely associated with infection count (Supplemental Fig. S3).

We next examined the subset of simulations that failed to maintain transmission to determine whether it was because Sabin 2 did not genetically revert. These die-outs were not due to incomplete Sabin 2 reversion and show that complete Sabin 2 genetic reversion does not guarantee stable transmission. Regardless of whether an epidemic failed, all simulated viral populations had infectiousness and shedding durations equivalent to or exceeding that of WPV within the first 200 days of transmission (Supplementary Fig. S2B, C). These results highlight the importance of epidemiological factors, such as the local immunity and contact structure present in our multiscale transmission model, which play a critical role in determining sustained transmission and outbreak probability.

### Population-level immunity and hygiene are major constraints of cVDPV2 emergence

**Figure 5.**
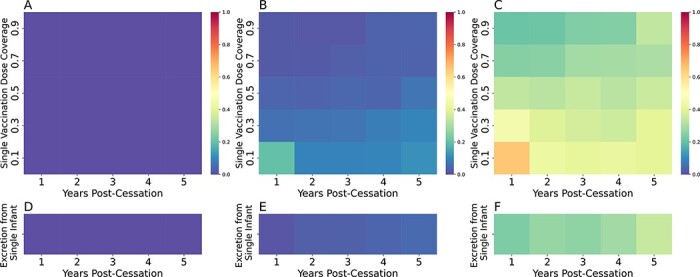
cVDPV2 outbreak risk. cVDPV2 emergence after an mOPV2 vaccination campaign (A–C) or importation of a single infant immunized with Sabin 2 (D–F) occurring up to 5 years of vaccination cessation with fecal–oral doses equivalent to Matlab (4.0e-7 grams per contact, A/D), five times greater (2.0e-6 grams per contact, B/E), and ten times greater (4.0e-6 grams per contact, C/F) than that inferred for Matlab during an mOPV2 clinical trial. Each cell in the heatmap represents results from 800 simulation runs.

We next utilized this model to simulate cVDPV2 emergence and understand risk. Our scenarios included a point importation of an infant vaccinated with Sabin 2 and a mass vaccination campaign with up to 90 per cent coverage in children under 5 years of age. We explored scenarios with fecal–oral doses equal to, five times greater, and ten times greater than Matlab (Fig. 5). The fecal–oral dose, combined with the infection-specific, time-dependent viral shedding concentration per gram of stool (Famulare et al. 2018), determines the total exposure dose per infectious contact. cVDPV2 emergence was defined as an outbreak significant enough to trigger a warning from passive routine surveillance efforts. We define cVDPV2 emergence in this paper as a simulation with sustained cVDPV2 transmission for at least 3 years. This was to separate scenarios resulting in endemic transmission from transient outbreaks where the transmission will die out with no intervention. As such, these results are not directly analogous to outbreaks that are defined epidemiologically, as it is unknowable to determine whether an observed outbreak is transient or endemic when an epidemiological outbreak is first defined.

We found zero risk of cVDPV2 emergence following a mass vaccination campaign (Fig. 5A) or point importation event (Fig. 5D) in Matlab with its current fecal–oral dose. However, cVDPV2 emergence is possible at higher fecal–oral doses. These higher fecal–oral dose simulations show that high vaccine coverage is essential for controlling cVDPV2 outbreak risk and that cVDPV2 outbreak risk is increasing over time due to the Switch. Broadly speaking, low-coverage, single-dose vaccination campaigns have the highest risk of resulting in a cVDPV2 outbreak, and those that occurred the first year following cessation had the highest level of outbreak risk. Surprisingly, outbreak risk fell after the first year of cessation but gradually increased with each additional year.

To determine why outbreak risk was highest in the first year following cessation, we examined the number of infections and changes to population immunity during a 10 per cent coverage campaign occurring 1 and 5 years after cessation (Supplementary Fig. S4). As expected, prior to any vaccination campaign, population-level immunity 5 years after cessation was lower than that 1 year after cessation (Supplemental Fig. S4B–D). As a result, the number of VDPV infections caused by the vaccination campaign occurring 5 years after cessation resulted in a greater number of infections that resulted in faster boosting of population-level immunity (Supplementary Fig. S4A). In other words, the high population-level immunity 1 year after cessation limited the number of VDPV infections during the initial wave of infections (first 200 days) and limited the boosting of pre-existing immunity in the population. As a result, population-level immunity was not high enough to prevent the second wave of infections occurring due to residual transmission that survives the first 200 days (Fig. 4A). This scenario highlights the cyclical nature of cVDPV2 outbreaks and the need to rapidly induce population-level immunity in the first wave of infections to prevent residual transmission from seeding new outbreaks.

After the first year of cessation, cVDPV2 outbreak risk fell but gradually rose with increased time since cessation and declining vaccination coverage. When the fecal–oral dose was 2.0e-6 grams per contact, five times that inferred for Matlab (4.0e-7 grams per contact), cVDPV2 emergence risk was less than 0.10 in all examined scenarios with two or more years post cessation (Fig. 5B–E). When the fecal–oral dose was ten times that of Matlab (4.0e-6 grams per contact), cVDPV2 emergence rose significantly, ranging from 0.20 to 0.40 in all examined scenarios occurring two or more years after cessation (Fig. 5B). cVDPV2 risk from point importation was also significant, and nearly a third of point importations resulted in a cVDPV2 outbreak (Fig. 5F).

## Discussion

cVDPV2 outbreaks are serious public health dilemmas made possible by viral evolution and exacerbated by epidemiological conditions. Our previous work simulated the Sabin 2 vaccine virus spread in the absence of genetic reversion. The primary goal of this work was to re-assess how Sabin 2 vaccine virus spreads as it mutates and phenotypically behaves more like WPV. This study combined data from multiple sources to develop an evolutionary epidemiology model of Sabin 2 genetic reversion and transmission that was then applied to specifically examine Sabin 2 cVDPV2 emergence risk in Matlab, Bangladesh. Focusing our simulations on Matlab, Bangladesh, allowed us to characterize how cVDPV2 reversion emerges and spreads in a setting where population-level immunity and demographic structure were previously parameterized (Famulare et al. 2021).

Our work contributes to the large body of poliovirus modeling work that has been used to assess poliovirus transmission risk (reviewed in Thompson and Kalkowska 2020). By expanding a previously calibrated, multiscale, Sabin 2 vaccine virus transmission model, we generated estimates of cVDPV2 outbreak risk in simulated populations that incorporate the differential transmission rates arising from household and demographic structure in real populations. Accurately capturing these differential transmission rates was required to replicate vaccine-derived Sabin 2 vaccine shedding in Matlab, Bangladesh (Famulare et al. 2021). We previously found that failing to account for these differential transmission rates risked overestimating transmission and outbreak risk.

Sabin 2 reversion can be viewed as an adaptive process where Sabin 2 must climb up a fitness landscape to achieve the optimal, WPV-like phenotype. To overcome the lack of direct transmission measurements for Sabin 2 reversion intermediates, we generated genotype-to-phenotype maps for shedding duration and viral infectiousness by relying on established molecular evolution and population genetic theory. A core feature of our model is that we assume that mutations that fixate shortly after vaccination have larger fitness effects than those that appear later.

This is loosely based on Fisher’s Geometric Model of adaptive evolution, which predicts that the variance in the DOFEs depends on the position on the fitness landscape (Hartl and Taubes 1998; Tenaillon 2014); the further away one is from the fitness optima, the larger the variance (and thus the range of tolerated phenotypic effects) in the DOFE. For the gatekeeper mutations, we assume A481G confers the greatest increase in shedding duration and infectiousness, while U398C confers the smallest. For the non-gatekeeper nonsynonymous mutations, we assume that early adaptive evolution (prior to the fixation of the three gatekeeper mutations) is dominated by deleterious nonsynonymous mutations, while long-term adaptive evolution (after the fixation of the three gatekeepers) is dominated by neutral nonsynonymous mutations (Stern et al. 2017).

Our model does not include later reversion events (the 5ʹ and 3ʹ recombinations with human enterovirus C (Xiao et al. 2016), U2523C, C2006A, U1376A, U1376A, and U3320A). These mutations are not as ubiquitous as the gatekeeper mutations but are hypothesized to represent smaller changes that fine-tune viral fitness in the weeks and months after the fixation of the three gatekeeper mutations. As such, our model may overestimate the rate with which Sabin 2 reverts and cVDPV2 emerges. Re-calibrating our model to include these later reversion events could ease the overshoot in viral infectiousness observed during the first 200 days in our simulations (Fig. 3D) by redistributing a small portion of the infectiousness gain across the 30 months needed to acquire the final mutation in the expanded evolutionary pathway (U3320A). Thus, our model predictions represent a worst-case scenario that attributes the majority of Sabin 2 reversion and cVDPV2 to the emergence of the three gatekeeper mutations.

Another potential limitation of our model is that we assumed that the three gatekeeper mutations revert independently and did not include any conditional or epistatic effects that could influence gatekeeper mutation reversion order. It is unclear how strict this order is due in part to the limited timescale of emergence and fixation of these mutations; these mutations fixate on the order of days to weeks and all three can be reverted within the vaccinated individual. There is some evidence to support the independent emergence of these gatekeeper mutations from the recent novel, serotype 2, oral poliovirus vaccines (nOPV2). In nOPV2, the stabilization of A481 does not prevent mutations at the domain-IV nucleotide 459 hairpin site, which is homologous to U398 in Sabin 2. It is unlikely that this assumption has a large effect on our predictions, as ∼0.887 of the viruses excreted from simulated Sabin 2–vaccinated individuals were consistent with the canonical evolutionary pathway described in Stern et al. (Supplemental Table S1). However, whole-scale recombination of the 5ʹ-region with a different human enterovirus or an already reverted Sabin 2 strain could cause cVDPV2 emergence to emerge faster than simulated here.

Interestingly, we failed to identify a deleterious cost associated with nonsynonymous mutations and shedding duration but did identify the one associated with nonsynonymous mutations and infectivity. This finding suggests that there are differences between intra-host and inter-host selection, which is supported by the elevated rates of nonsynonymous mutation accumulation in vaccinated individuals as compared to the rates observed in individuals infected with circulating, vaccine-derived virus (Supplementary Fig. S1, Famulare et al. 2015). Biologically, intra-host selection is mediated by the immune system (Dunn et al. 2015), and strong immune-drive purifying selection could explain why we were unable to identify a fitness cost associated with nonsynonymous mutation accumulation and shedding duration. Future studies quantifying the effect of immune-driven purifying selection between immunocompromised and immunocompetent individuals (Dunn et al. 2015) would be useful for better understanding the effects of intra-host selection on poliovirus evolution. Conversely, the efficacy of inter-host selection (infectiousness) may be weakened by the severe sampling of viral genotypes during person-to-person transmission (Hughes 2008; McCrone et al. 2018). Limited sampling enhances the effects of genetic drift and could make it difficult to efficiently remove deleterious mutations (Hughes 2008).

Despite the emergence of WPV-like viruses in our simulations, complete genetic reversion of Sabin 2 does not guarantee a cVDPV2 outbreak. Population-level immunity, exposure, and transmission heterogeneity all play a significant role in determining whether a cVDPV2 outbreak occurs (McCarthy et al. 2017; Famulare et al. 2018; Jorba et al. 2019; Macklin et al. 2020; Thompson and Kalkowska 2020). For Matlab, Bangladesh, the modeled household community and contact structure likely influenced outbreak dynamics and limited the spread of the revertant virus despite complete genetic reversion. The interplay between genetics and epidemiology lends itself to a complex outbreak risk landscape that can lead to unexpected and unintuitive results. One such example is our prediction that the highest cVDPV2 outbreak risk in Matlab was following a low vaccination campaign in the year immediately following cessation, which we suspect is driven in part by the unique immunity structure of the population at the time. Despite the rapid reversion of the Sabin 2 vaccine virus, cVDPV2 emergence risk is still constrained by the availability of susceptible hosts and the amount of population-level immune boosting immediately following an mOPV2 campaign.

The strategies for controlling cVDPV2 involve implementing aggressive OPV2 SIA activities in outbreak response zones and neighboring regions, intensifying routine immunization in high-risk areas with inactivated polio vaccine, ensuring a sufficient supply of OPV2 for future eradication strategies, and identifying high-risk zones (Geneva: World Health Organization 2020). However, ensuring that these interventions are implemented at the standard needed to contain vaccine virus transmission and prevent cVDPV2 emergence can be difficult. Due to its potential to genetically revert, OPV2 use must be carefully monitored to mitigate cVDPV2 emergence risk. With the development of nOPV2, there is much optimism that rates of reversion and emergence will decline, but genetic reversion is still possible using this vaccine (Yeh et al. 2020; Wahid et al. 2021), and its behavior in a constantly changing immunity landscape is currently being evaluated.

For Matlab, we show that cVDPV2 outbreaks can still occur if the fecal–oral dose is too high. This parameter determines the average exposure to infectious viral particles and depends on both the concentration of viral particles per gram of fecal material shed by an infected individual and the average fecal–oral dose per contact. One way of interpreting this parameter is as a proxy for the overall fecal sanitation in the region. These results suggest any intervention aimed at reducing exposure, such as during a hygienic campaign aimed at improving sanitation levels so that people are less frequently exposed to fecal matter, could help mitigate cVDPV2 emergence risk. One potential way that the cVDPV2 emergence risk can be mitigated is by sanitation assessments in both the targeted intervention zone and the surrounding regions. These assessments could use tools to measure fecal contamination of other fecal–oral pathogens such as *Escherichia coli* (Wang et al. 2017) and be useful for resource allocation and for determining the minimum coverage needed to prevent future cVDPV2 outbreaks since regions with poor sanitation require more effective SIA vaccination campaigns to prevent seeding future cVDPV2 outbreaks when using mOPV2.

Our model lays the foundations for integrating population genetic theory and epidemiology to study the evolutionary epidemiology dynamics of rapidly evolving infectious diseases. The success of our framework hinges on an understanding of a pathogen’s evolutionary trajectory as well as the initial and ending phenotypic states. We anticipate that our model will be extended to the nOPV2 candidates (Van Damme et al. 2019) as more about their clinical phenotypes and molecular evolution are known. Depending on how attenuated and evolutionarily stable the nOPV2 candidates are relative to Sabin 2, mass nOPV2 vaccination may reduce the need to also control sanitation levels. We also anticipate that our model will be useful for assessing the risks associated with the live-attenuated varicella-zoster vaccines, which also shows strong evidence of attenuation reversion conferred by a commonly accessed evolutionary pathway (Breuer 2018).

## Materials and methods

### Sequencing data

We downloaded FASTA files for the VP1 segments and whole genome sequences of Sabin 2 and Sabin 2–derived genomes from GenBank. These samples were collected across 102 studies performed in countries including Egypt, Madagascar, China, Nigeria, the USA, Israel, Switzerland, and the Democratic Republic of Congo. Multiple sequence alignments were first generated using MUSCLE (v3.8.31) (Edgar 2004). Sequences were compared to a known Sabin 2 sample (accession ID: AY184220) to identify and remove insertions. Sequences with long internal gaps (more than fifteen bases) and low coverage <30 per cent (percentage of called bases) were discarded. Coding sequence alignments from the whole genome sequences were generated for each of the poliovirus coding regions by generating multiple sequence alignments for each of the fifteen translated protein-coding sequences of the poliovirus genome. These alignments were further filtered to remove samples with <90 per cent coding sequence coverage.

A total of 177 whole genome sequences and 1,643 VP1 sequences were retained for analysis. Samples were classified as Sabin 2, Sabin-like, VDPV, iVDPV (immunodeficiency-related VDPV), and cVDPV2 based on their original study classifications. Viruses that displayed <1 per cent VP1 nucleotide sequence difference from the parental Sabin 2 genome were classified as Sabin-like and those with 1–15 per cent nucleotide sequence difference were classified as VDPV. VDPVs isolated from immunodeficient individuals were classified as iVDPV, while samples with evidence of having originated from prior transmission are classified as cVDPV2. The whole genome sample set also included the Lansing and MEF-1 laboratory strains as well as several engineered Lansing strain derivatives. Accession numbers, classifications, and study origin for each sample are given in Supplemental File 1 and Supplemental File 2.

Coding sequence alignments were used to generate a phylogenetic tree using RAxML (v8.2.10) with a general time-reversible CAT (GTRCAT) model (Stamatakis 2014). Estimates of nonsynonymous and synonymous mutation counts and dN/dS were based on the RAxML-generated phylogenetic tree. Estimates of nonsynonymous and synonymous mutation counts from the VP1 sequences were counted directly from the sequence data. Nonsynonymous and synonymous mutation counts for codons with multiple mutations were averaged across all possible, unweighted evolutionary paths.

## Model description

### Multiscale transmission model

Overall, transmission is modeled in populations with evolving household and demographic structure. Individuals are grouped into different households, *baris* (clusters of related households analogous to a neighborhood), and villages. Individuals are born into households whose composition changes based on the local fertility rates, mortality rates, and an anthropological framework for describing household structure and evolution in rural Bangladeshi society. Household and demographic structure are an essential part of the model and are used to inform heterogeneous contact rates to household, *bari*, village, and non-village members.

Each agent in the model represents an individual whose infection dynamics are controlled by immunity and specified by an OPV-equivalent antibody rate. Immunity is individual specific and is critical for determining the susceptibility and transmission potential of individuals. For infected individuals, immunity controls the shedding duration and viral shedding titer, which is defined as the number of infectious viral particles per gram of fecal matter. Immunity also determines the shedding duration and viral shedding concentration of infected individuals; individuals with low immunity prior to infection have longer shedding durations and lower viral shedding titers. These were previously calibrated to data from historical vaccine trials and WPV exposures (Famulare et al. 2018).

Transmission occurs when an infected individual contacts and transmits an infectious virus to other individuals. Each infected individual makes a different number of daily contacts to household, *bari*, and village members. During each contact, infected individuals expose others to a model-inferred fecal–oral dose that describes the total amount of fecal material that a recipient will receive during each contact event. The amount of infectious virus is then determined by multiplying the fecal–oral dose with the viral shedding titer of the infectious individual at the time of contact. Whether the recipient individual is infected depends on their susceptibility, which is defined by a dose–response model that determines the probability of infection given the strain-specific viral infectiousness, the total viral exposure dose per contact, and the immunity of the recipient host. Upon successful infection or vaccination, the OPV-equivalent antibody titer increases based on the OPV-equivalent antibody titer at the time of infection or vaccination. In the absence of further exposure, this antibody titer wanes over time. Complete, mathematical descriptions of the individual infection dynamics and the multiscale transmission model were described elsewhere (Famulare et al. 2018, 2021). Previous parameterizations of the individual infection dynamics assumed stable viral phenotypes whose shedding duration and viral infectiousness were constant over time. When incorporating genetic reversion, we assumed that the equations governing immunity, shedding duration, and susceptibility followed the same mathematical forms. When incorporating genetic reversion in these models, we focused on re-calibrating the shedding duration and infectiousness equations described in Famulare et al. (2018), so that these values change during the course of Sabin 2 genetic reversion (see later; Materials and methods; Genotype to phenotype: Mapping genetics to shedding duration and infectiousness; and SI Appendix). When re-calibrating the multiscale transmission model, we assumed that the contact structure remained the same and focused on re-calibrating the fecal–oral dose (SI Appendix).

### Defining genotypes

Genotypes are defined relative to the Sabin 2 genome and defined by the reversion state of three gatekeeper mutations (A481G, U2909C, and U398C) and the number of newly derived nonsynonymous and synonymous mutations in the rest of the genome. The Sabin 2 genome is defined as an array with six different mutation classes:


$${G_{S2}} = \left[ {0,\,0,\,0,\,0,\,0,0} \right]$$

The first three are Boolean flags that indicate the allelic states of A481G, U2909C, and U398C (0 = unreverted and 1 = reverted). The last three elements represent the total number of deleterious nonsynonymous, neutral nonsynonymous, and synonymous mutations present in the genome. The counts in the latter categories exclude the three gatekeeper mutations. Splitting nonsynonymous mutations into neutral and deleterious simplifies the true DOFEs and was necessary to reconcile phenotypic and molecular evolution (SI Appendix). These genotypes represent the consensus sequence of fixed variants within vaccinated and infected individuals.

For evolved poliovirus strains, we use the notation:


$${G_{i,j,k,nonsy{n_{del}},\,nonsy{n_{neutral}},syn}}$$

where *i, j*, and *k* are replaced with the allelic states of A481G, U209C, and U398C. $nonsy{n_{neutral}}$, $nonsy{n_{del}}$, and $syn$ are replaced with their respective counts or replaced with *X* if these mutation classes are irrelevant. For example, ${G_{0,0,0,0,0,0}}$ is equivalent to the unmutated Sabin 2 vaccine strain (${G_{S2}}$) and ${G_{1,1,1,10,2,15}}$ is a genotype where all three gatekeeper mutations have reverted and where ten deleterious nonsynonymous, two neutral nonsynonymous, and fifteen synonymous mutations have accumulated.

### Genomic evolution: Modeling Sabin 2 molecular evolution

Genomic evolution is modeled using intra-host substitution rates (λ), the fixation of newly derived mutations within the host, for each mutation class (${\lambda _{A481G}}$, ${\lambda _{U2909C}}$, ${\lambda _{U398C}}$, ${\lambda _{syn}}$, ${\lambda _{nonsyn,\,del}}$, and $\,{\lambda _{nonsyn,\,neutral}}$). This substitution rate reflects the true de novo mutation rate, genetic drift, and selection within the host. This simplification allows us to use a single consensus genome to represent the fixed mutations present in the intra-host viral population. Each mutation class (the three gatekeeper mutations, synonymous mutations, deleterious nonsynonymous mutations, and neutral nonsynonymous mutations) is associated with a substitution rate that describes its expected fixation time (Supplemental File 3).

To accommodate the effects of intra-host competition and recombination, deleterious nonsynonymous mutation accumulation also depends on a recombination-like mechanism with rate ${\lambda _{recombination}}$ that purges deleterious mutations from the genome (Chao 1990; Bessaud et al. 2016; Xiao et al. 2016). Intra-host substitution rates for the gatekeeper mutations were used as described in Famulare et al. (2015), while ${\lambda _{syn}}$, ${\lambda _{nonsyn,\,del}}$,$\,{\lambda _{nonsyn,\,neutral}}$, and ${\lambda _{recombination}}$ were calibrated to the whole genome sequences and VP1 segments downloaded from GenBank. Only confirmed Sabin 2, Sabin-like, VDPV, and cVDPV samples were used for calibration. All other samples were excluded from calibration. Calibration details are provided in the SI Appendix.

Upon vaccination with Sabin 2, fixation times for all mutation classes are calculated by drawing from exponential distributions whose rate parameters are defined by the substitution rate of each mutation class. For the three gatekeeper mutations, these fixation times determine the time to reversion. For nonsynonymous and synonymous mutations, these fixation times determine the time to the next substitution event. To prevent Mueller’s Ratchet from resulting in an infinite deleterious mutation load, we also draw a time to the next ‘recombination’ event from an exponential distribution defined by ${\lambda _{recombination}}$. During each ‘recombination’ event, the model draws a random number from a discrete, uniform distribution ranging from zero to ${n_{nonsyn,deleterious}}$, the number of deleterious nonsynonymous mutations in the genome, to determine the number of deleterious mutations to remove. Conceptually, this represents purifying selection within the host and allows us to accommodate the assumed increase in purifying selection as Sabin 2 reverts to cVDPV2. Pseudocode is provided in Supplementary Fig. S5.

### Genotype to phenotype: Mapping molecular genetics to shedding duration and infectiousness

A core assumption of our model is that each of the three gatekeeper mutations contributes to the final, WPV phenotype proportional to their relative substitution rates. This assumption is based in part on evolutionary fitness landscape and adaptation theory, which posits that beneficial mutations of large effect are more likely to be fixed early on when an organism is far the optimal fitness peak on a simple, non-rugged fitness landscape (Svensson and Calsbeek 2012; Tenaillon 2014). By definition, attenuated Sabin 2 vaccine strains are far from the optimal fitness peak for human infection, and genetic reversion is the process of climbing the fitness landscape to reach the optimal, WPV-like phenotype. Because of their fast substitution rate, we attribute the bulk of phenotypic change to A481G, followed by U2909C, and to a lesser extent U398C. The ranked ordering of phenotypic effect in our model is supported in the literature. A481G and U2909C are consistently identified as the primary molecular determinants of reversion in *in vitro* and *in vivo* studies (Muzychenko et al. 1991; Ren, Moss, and Racaniello 1991; Simoes and Sarnow 1991; Kew et al. 2005; Duintjer Tebbens et al. 2013b; Cassemiro et al. 2016). U398C is only occasionally identified by these studies, but its recurrent fixation within the first few months of transmission (Famulare et al. 2015; Stern et al. 2017) suggests that its adaptive benefit may be too small to be assessed in laboratory-based assays relative to A481G and U2909C.

Two epidemiologically relevant phenotypes were calculated for each infection based on its corresponding genotype: (1) shedding duration and (2) infectiousness. Both shedding duration and infectiousness are based on a previously described poliovirus dose–response model (Famulare et al. 2018). Conceptually, the shedding duration is analogous to intra-host selection and infectiousness is analogous to inter-host selection. Details regarding model calibration are deferred to the SI Appendix.

Shedding duration is correlated with pre-challenge immunity (Famulare et al. 2018). Our modeled shedding duration is designed to replicate this correlation and include individual-level randomness using a log-normal duration distribution with pre-challenge immunity-dependent median with the form:


(1) $$ & P\left( {shedding}\, {at}\, t\, {\mathrm{|}}{N_{a{b_{pre}}}};{infected}\, {at}\, t = 0 \right) \nonumber\\
&\qquad= \frac{1}{2}\left( {1 - erf\left( {\frac{{\ln \left( t \right){\mkern 1mu} - {\mkern 1mu} ({{\mathrm{M}}_{\mathrm{g}}}{\mkern 1mu} - {\mathrm{log}}\left( \delta \right){{\log }_2}({N_{a{b_{{\mathrm{pre}}}}}})}}{{\sqrt 2 \ln \left( {{{\mathrm{S}}_{\mathrm{g}}}} \right)}}} \right)} \right)$$

where ${N_{a{b_{pre}}}}$ is the pre-infection OPV-equivalent antibody titer and $\delta $ is the median reduction in shedding duration per log(${N_{a{b_{pre}}}}$) (Famulare et al. 2018). For each genotype, ${{\mathrm{M}}_{\mathrm{g}}}$ and ${S_g}$ are genome-specific parameters that influence the average shedding duration and its standard deviation. The mean of this log-normal distribution is defined by ${{\mathrm{M}}_{\mathrm{g}}}\, - {\mathrm{log}}\left( \delta \right){\log _2}({N_{a{b_{pre}}}})$ and its standard deviation is defined by ${{\mathrm{S}}_{\mathrm{g}}}$.


(2) $${M_g} = \log \left( {{\mu _{S2}}} \right) + {w_{{shedding}, g}}$$

(3) $${S_g} = \sigma \sqrt {\left| {1 + {w_{{shedding}, g}}} \right|} $$

(4) $${w_{{shedding}, g}} =\,& 0 + {\mkern 1mu} {s_{{\mathrm{dur}}, {\mathrm{A481G}}}}*{G_{{\mathrm{A481G}}}} + {s_{{\mathrm{dur,U2909C}}}}*{G_{{\mathrm{U2909C}}}} \nonumber\\&+ {s_{{\mathrm{dur,U398C}}}}*{G_{{\mathrm{U398C}}}} + {s_{{\mathrm{dur,nonsy}}{{\mathrm{n}}_{{\mathrm{del}}}} }}*{n_{{\mathrm{nonsy}}{{\mathrm{n}}_{{\mathrm{del}}}}}}$$

where ${\mu _{S2}}$ is the average shedding duration of a completely unevolved Sabin 2 virus (${G_{S2}}$) in immunologically naive individuals (${N_{a{b_{pre}}}} = 1)$ and$\,\sigma $ is the standard deviation in shedding duration for ${G_{S2}}$. ${w_{shedding,\,g}}$ defines the shedding duration fitness of a strain; ${G_{A481G}}$, ${G_{U2909C}}$, and ${G_{U398C}}$ refer to the allelic status of the three gatekeeper mutations; and ${s_{dur,A481G}}$, ${s_{dur,U2909C}}$, and ${s_{dur,U398C}}$ are selection coefficients that describe the change in shedding duration conferred by each of these mutations. Note that fitness is additive but in log space.

Upon infection or vaccination, we randomly draw a shedding duration from a log-normal distribution with mean ${{\mathrm{M}}_{\mathrm{g}}}\, - {\mathrm{log}}\left( \delta \right){\log _2}({N_{a{b_{pre}}}})$ and standard deviation ${{\mathrm{S}}_{\mathrm{g}}}$. A core feature of our shedding duration model is that new gatekeeper mutations arising within the host extend shedding duration. If a new gatekeeper mutation is acquired, the shedding duration is extended:


(5) $${\mathrm{Duration}} = {\mathrm{Duratio}}{{\mathrm{n}}_{{\mathrm{initial}}}}*{e^{{S_{{\mathrm{gateway}}}} + {\mathrm{Norm}}\left( {0,\sigma } \right)\hbox{$\scriptsize\sqrt{{}^{S_{{\rm gateway}}}}$} }}$$

(6) $${S_{{\mathrm{gateway}}}}{\mkern 1mu} \in \left\{ {{s_{dur,A481G}},{\mkern 1mu} {s_{dur,U2909C}},{\mkern 1mu} {s_{dur,U398C}}{\mkern 1mu} } \right\}$$

where ${D_{initial}}$ is the shedding duration before acquisition of the gatekeeper mutation and ${S_{gateway}}$ is the shedding duration selection coefficient for the gatekeeper mutation. Note that $Duratio{n_{initial}}$ and the increase in shedding duration, ${e^{{S_{gateway}}\, + \,Norm\left( {0,\sigma \,} \right)\hbox{$\scriptsize\sqrt{{}^{S_{{gateway}}}}$} }}$, are log-normally distributed and that multiplying two, independent log-normal distributions results in a log-normal distribution whose mean and variance are the sum of the mean and variances of the original distributions. As a result, shedding duration is the summation of multiple, genome-specific shedding durations whose weights are time dependent and depend on the fixation of A481G, U2909C, and U398C and the accumulation of nonsynonymous mutations.

Infectiousness is defined by a beta-Poisson model that assumes that a single infectious unit is sufficient to start an infection and that multiple infectious units contribute independently to the probability of infection:


(7) $$P\left( {{\mathrm{infection|dose}},{\mkern 1mu} {N_{ab}}} \right) = 1 - {\left( {1 + \frac{{{\mathrm{dose}}}}{{{\beta _g}}}} \right)^{ - \alpha {{\left( {{N_{ab}}} \right)}^{ - \gamma }}}}$$

where $\alpha \,$and ${\beta _g}$ are parameters for a beta-Poisson function, dose is the viral exposure dose, and $\gamma $ captures the reduction in shedding probability with increasing immunity. Altering the $\beta $ parameter shifts the beta-Poisson function along the x-axis (dose) and altering the $\alpha $ parameter changes its slope. Differences in infectiousness between Sabin 2 strains and WPV can be modeled using the same $\alpha $ but with modified $\beta $ parameters (Famulare et al. 2018). We interpret the $\beta $ parameter as a measurement of strain infectivity and define ${\beta _g}$ as follows:


(8) $$
{\beta _g} = \frac{\beta}{
\begin{array}{l}
{\left( {1 + {s_{inf},A481G}} \right)}^{G_{A481G}} {\left( {1 + {s_{inf}},U2909C}\right)}^{G_{U2909C}}\\
{\left( {1 + {s_{inf},U398C}} \right)}^{G_{U398C}} {\left( 1 + {s_{{inf},{nonsy} {n}_{del}}} \right)}^{n_{{nonsy}{n}_{del}}}
\end{array}}
$$

where $\beta $ is the strain infectivity parameter for Sabin 2 (Famulare et al. 2018) (assuming no evolution); ${s_{inf,A481G}}$, $\,{s_{inf,U2909C}}$,$\,\,{s_{inf,U398C}}$, and ${s_{inf,nonsy{n_{del}}}}$ are selection coefficients denoting the change in infectiousness conferred by each of the gatekeeper mutations; and ${n_{nonsy{n_{del}}}}$ are the number of nonsynonymous mutations in the genome. Parameter estimates were obtained by comparing the CCID50 between Sabin 2 and WPV (SI Appendix). The inferred selection coefficients for the three gatekeeper mutations are positive (increases infectiousness), while the selection coefficient for deleterious nonsynonymous mutations was negative (decreases infectiousness). Infectiousness is calculated only at the onset of either vaccination or infection.

## Supplementary Material

vead044_Supp

## Data Availability

Data and code are publicly available at https://github.com/InstituteforDiseaseModeling/cvpdv2-evo-epi.

## Classification

BIOLOGICAL SCIENCES: Evolution
