# Supplementary material for: From vaccine to pathogen: Modeling Sabin 2 vaccine virus reversion and evolutionary epidemiology in Matlab, Bangladesh
Source: Virus Evol. 2023 Jul 8;9(2):vead044. doi: 10.1093/ve/vead044 (PMC10491863; doi:10.1093/ve/vead044)
Supplement: vead044_Supp [file vead044_supp.zip › Supplemental Table 1.docx]

**Supplemental Table 1** Final viral genotypes in primary vaccine recipients and the expected shedding durations of individuals infected with these genotypes, assuming no further reversion. Bootstrapped confidence interval for the mean were calculated from 1000 simulated primary vaccinations, repeated 1000 times. Shedding durations are reported in days. Sabin 2 in this table is defined as a viral genotype with zero gatekeeper reversions.

| Genotype | Proportion | Expected Shedding Duration |
| --- | --- | --- |
| Sabin 2 (no reversions) | 0.076 (0.059, 0.093) | 14.39 (14.08, 14.69) |
| A481G only | 0.186 (0.162, 0.211) | 25.46 (24.71, 26.24) |
| U2909C only | 0.024 (0.014, 0.034) | 20.39 (19.85, 20.92) |
| U398C only | 0.01 (0.004, 0.016) | 18.98 (18.52, 19.47) |
| A481G + U398C | 0.075 (0.059, 0.091) | 33.53 (32.46, 34.62) |
| U398C + U2909C | 0.005 (0.001, 0.01) | 26.90 (26.10, 27.72) |
| A481G + U2909C | 0.273 (0.247, 0.300) | 36.08 (34.93, 37.26) |
| A481G + U2909C + U398C | 0.352 (0.325, 0.382) | 47.51 (45.93, 49.174) |
