# Supplementary material for: From vaccine to pathogen: Modeling Sabin 2 vaccine virus reversion and evolutionary epidemiology in Matlab, Bangladesh
Source: Virus Evol. 2023 Jul 8;9(2):vead044. doi: 10.1093/ve/vead044 (PMC10491863; doi:10.1093/ve/vead044)
Supplement: vead044_Supp [file vead044_supp.zip › Supplemental Table 2.docx]

**Supplemental Table 2 Intra-host substitution rates** (substitutions per base pair per day) All priors are based on VP1 segments collected in Nigeria. The mean posterior value is used as our point estimate. Posterior estimates for the three gatekeeper mutations are identical to the values reported in the Nigeria study (Famulare et al. 2015).

| **Parameter** | **Prior** | **Mean Posterior** | **Lower 95%** | **Upper 95%** |
| --- | --- | --- | --- | --- |
| λ_recomb_ | 3.62E-06 | 5.24E-06 | 3.13E-06 | 7.50E-06 |
| λ_nonsyn,del_ | 2.05E-05 | 1.83E-05 | 9.78E-06 | 3.02E-05 |
| λ_nonsyn,neutral_ | 3.32E-06 | 3.34E-06 | 2.93E-06 | 3.66E-06 |
| λ_syn_ | 3.00E-05 | 3.16E-05 | 3.02E-05 | 3.29E-05 |
|  |  |  |  |  |
| λ_A481G_ | 1.54E-01 | 1.54E-01 | 7.00E-02 | 2.97E-01 |
| λ_U2909C_ | 4.70E-02 | 4.70E-02 | 1.80E-02 | 9.60E-02 |
| λ_U398C_ | 2.10E-02 | 2.10E-02 | 6.00E-03 | 4.80E-02 |
