# Supplementary material for: From vaccine to pathogen: Modeling Sabin 2 vaccine virus reversion and evolutionary epidemiology in Matlab, Bangladesh
Source: Virus Evol. 2023 Jul 8;9(2):vead044. doi: 10.1093/ve/vead044 (PMC10491863; doi:10.1093/ve/vead044)
Supplement: vead044_Supp [file vead044_supp.zip › Supplemental Table 3.docx]

**Supplemental Table 3 Shedding duration parameters** assuming $s_{dur,nonsyn_{del}}$affected shedding duration. *δ* was not estimated for this version of the model.

| **Parameter** | **Prior** | **Posterior** | **Lower 95%** | **Upper 95%** |
| --- | --- | --- | --- | --- |
| μ | 12.000 | 13.160 | 11.001 | 15.394 |
| σ | 0.300 | 0.325 | 0.274 | 0.378 |
| s_dur,A481G_ | 0.600 | 0.574 | 0.389 | 0.757 |
| s_dur,U2909C_ | 0.367 | 0.351 | 0.238 | 0.463 |
| s_dur,U398C_ | 0.291 | 0.278 | 0.188 | 0.367 |
| *s_dur,nonsyn,del_* | *0.000* | *-0.006* | *-0.041* | *0.029* |
| δ |  |  |  |  |
