# Supplementary material for: From vaccine to pathogen: Modeling Sabin 2 vaccine virus reversion and evolutionary epidemiology in Matlab, Bangladesh
Source: Virus Evol. 2023 Jul 8;9(2):vead044. doi: 10.1093/ve/vead044 (PMC10491863; doi:10.1093/ve/vead044)
Supplement: vead044_Supp [file vead044_supp.zip › Supplemental Table 4.docx]

**Supplemental Table 4 Shedding duration parameters** assuming $s_{dur,nonsyn_{del}}$had no effect on shedding duration phenotype. These were the final parameter estimates used in our model.

| **Parameter** | **Prior** | **Posterior** | **Lower 95%** | **Upper 95%** |
| --- | --- | --- | --- | --- |
| μ | 12.000 | 13.790 | 11.990 | 14.410 |
| σ | 0.300 | 0.360 | 0.310 | 0.400 |
| s_dur,A481G_ | 0.600 | 0.535 | 0.460 | 0.660 |
| s_dur,U2909C_ | 0.367 | 0.327 | 0.281 | 0.404 |
| s_dur,U398C_ | 0.291 | 0.259 | 0.223 | 0.320 |
| s_dur,nonsyn,del_ | 0.000 | -0.050 | -0.080 | -0.012 |
