# Supplementary material for: From vaccine to pathogen: Modeling Sabin 2 vaccine virus reversion and evolutionary epidemiology in Matlab, Bangladesh
Source: Virus Evol. 2023 Jul 8;9(2):vead044. doi: 10.1093/ve/vead044 (PMC10491863; doi:10.1093/ve/vead044)
Supplement: vead044_Supp [file vead044_supp.zip › Supplemental Table 5.docx]

**Supplemental Table 5 Infectiousness parameters**. β_wpv_ and β_S2_ were obtained from (Famulare et al. 2018).

| **Parameter** | **Point Estimate** |
| --- | --- |
| β_wpv_ | 2.30 |
| β | 8.00 |
| s_inf,A481G_ | 1.82 |
| s_inf,U2909C_ | 0.56 |
| s_inf,U398C_ | 0.25 |
| s_inf,nonsyn,del_ | -0.05 |
