# Supplementary material for: From vaccine to pathogen: Modeling Sabin 2 vaccine virus reversion and evolutionary epidemiology in Matlab, Bangladesh
Source: Virus Evol. 2023 Jul 8;9(2):vead044. doi: 10.1093/ve/vead044 (PMC10491863; doi:10.1093/ve/vead044)
Supplement: vead044_Supp [file vead044_supp.zip › suppl_data/cvdpv_ms_supplement_virus_evolution_revision3.docx]

Supplemental Information for

**From Vaccine to Pathogen: Modeling Sabin 2 Vaccine Virus Reversion and Evolutionary Epidemiology**

Wesley Wong^1^, Jillian Gauld^2^, Michael Famulare^2^

**Author Affiliations**

1. Harvard School of Public Health, Cambridge MA, USA
2. Institute for Disease Modeling, Global Health, Bill & Melinda Gates Foundation, Seattle, WA, USA.

**Corresponding Author**

Michael Famulare

500 5^th^ Ave N, Seattle, WA 98109

+1 (425) 691-3327

[mike.famulare@gatesfoundation.org](mailto:mike.famulare@gatesfoundation.org)

**Supplemental Text**

*Calibrating Sabin 2 molecular evolution*

Simulated Sabin 2 molecular is based on the reversion of three gateway mutations (A481G, U2909C, U398C) as well as the accumulation of nonsynonymous mutations (deleterious and neutral) and synonymous mutations. Intra-host substitution rates (λ) were previously calculated from 241 VP1 segments from Type 2 Sabin-like poliovirus collected during routine acute flaccid paralysis surveillance in Nigeria from 2009-2012 (Wassilak et al. 2011; Famulare et al. 2015). These estimates were obtained by counting the frequency of the three gateway mutations and examining the accumulation of nonsynonymous and synonymous mutations.

We used the average, posterior intra-host substitution rates reported in the Nigeria study (Famulare et al. 2015) for the three gateway mutations as their respective point estimates for intra-host substitution rates in our study ($\lambda_{A481G}$, $\lambda_{U2909C}$, $\lambda_{U398C}$) (**Supplemental Table 2**). The synonymous substitution rate calculated from the Nigerian dataset (Famulare et al. 2015) were not significantly different from those estimated from wild poliovirus (Jorba et al. 2008). However, the nonsynonymous substitution rates inferred from the Nigerian dataset were two orders of magnitude higher than from WPV. This difference likely reflects a change in selection pressure as Sabin 2 due to changing epidemic circumstances and as the virus phenotypically approaches WPV. As a result, we could not directly use the nonsynonymous substitution rates reported from the Nigerian dataset.

*Nonsynonymous mutations: Neutral and Deleterious*

We divided the distribution of fitness effects into two categories, deleterious and neutral, and assign each with a separate intra-host substitution rate, $\lambda_{nonsyn, del}$ and $\lambda_{nonsyn, neutral}$. These rates were calibrated to the Sabin 2, Sabin 2-like, and cVDPV2 whole genome sequences and VP1 segments downloaded from Genbank (**Methods**). These sequences include the ones collected in the Nigerian study (Famulare et al. 2015) but also include sequences reported to have been circulating for up to twenty years. Our goal was to infer $\lambda_{nonsyn, del}$, $\lambda_{nonsyn, neutral}$, $\lambda_{recombination}$, and $\lambda_{syn}$ based on the observed number of nonsynonymous mutations in the whole genome samples, the observed number of synonymous and nonsynonymous mutations in the VP1 segment samples (Famulare et al. 2015).

We simulated Sabin 2 molecular evolution in the absence of transmission to calibrate these parameters. During each simulation, 1000 Sabin 2 genomes were evolved independently in daily time steps for twenty years. Mutation accumulation and reversion was modeled by drawing from an exponential distribution with the corresponding intra-host substitution rates. The average number of nonsynonymous mutations (deleterious and neutral) were recorded at each time step and compared to the data using a Poisson pseudo-log-likelihood function:

| $L= \sum_{i}^{n_{wgs}} (\hat{x}_{wgs,t_{i}}+\log\left( x_{i}! \right)-x_{i}log(\hat{x}_{wgs,t_{i}}))+ \sum_{i}^{n_{vp1}} (\frac{903}{7400}\hat{y}_{wgs,t_{i}}+\log\left( y_{i}! \right)-y_{j}log(\frac{903}{7400}\hat{y}_{wgs,t_{i}}))$ | (S1) |
| --- | --- |

where $\hat{x}_{wgs,t_{i}}$ is the average number of nonsynonymous mutations in the simulated whole genome sequences at timepoint $t_{i}$, the time since vaccination for a given sample, $x_{i}$ is the number of nonsynonymous mutations observed for a given whole genome sequence,$\hat{y}_{wgs,t_{i}}$ is the average number of mutations (nonsynonymous and synonymous) mutations in the simulated VP1 segments at timepoint $t_{i}$, $y_{i}$ is the observed number of mutations a given VP1 segment, $n_{wgs}$ is the number of samples with whole genome sequences, and $n_{vp1}$ is the number of samples with vp1 sequence. Note that we assume that genome-wide mutation rates are the same in the VP1 segment, allowing us to calculate simulated VP1 mutations by multiplying $\hat{y}_{wgs,t_{i}}$ by the proportion of the genome within the VP1 segment. $t_{i}$ for each sample was calculated by dividing the observed number of synonymous mutations by $\lambda_{syn}$.

Posteriors for $\lambda_{nonsyn, del}$, $\lambda_{nonsyn, neutral}$, $\lambda_{recombination}$, and $\lambda_{syn}$ were generated using an Approximate Bayesian Computation - Sequential Monte Carlo (ABC-SMC) (Sadegh and Vrugt 2014) with 300 particles and three iterations. Each particle tests a parameter set by independently running the simulation described above. Priors for$\lambda_{syn}$ , $\lambda_{nonsyn, del}$ and $\lambda_{nonsyn, neutral}$ were based on the Nigerian nonsynonymous substitution rates and the WPV nonsynonymous substitution rates, respectively (Famulare et al. 2015). A prior for $\lambda_{recombination}$ was obtained by performing a parameter sweep using the priors for the other three parameters to identify a value that approximates the nonsynonymous mutations and VP1 mutations from samples collected within one year of vaccination (**Supplemental Figure 6**). Error thresholds for each iteration were empirically generated by drawing 1000 parameter sets from our priors and set at the 30^th^, 10^th^, and 5^th^ percentiles. Priors and the final posterior estimates are reported in **Supplemental Table 2**. The mean posterior value was used as our point estimates for all other simulations.

*Genotype to Phenotype: Calibrating shedding duration*

*Identifying selection coefficients and the base shedding duration*

The average shedding duration for poliovirus is lognormally distributed, with a mean and median of 38.3 and 30.3 days for Sabin 2 vaccine recipients and 48 and 43 days during WPV infection in immunologically naïve individuals (Famulare et al. 2018). Here, we attribute the increase in shedding duration to the reversion of the three gateway mutations and a small number of deleterious nonsynonymous mutations. Our goal was to replicate the change in shedding duration from Sabin 2 and WPV as Sabin 2 evolves using **Equations 1-8**. We also utilized a modified form of **Equation 5**:

| $Duration={Duration}_{initial}*\left( e^{S_{dur, nonsyn} + Norm\left( 0,\sigma\right)\sqrt{s_{dur, nonsyn}}} \right)^{\Delta_{nonsyn,del}}$ | (S2) |
| --- | --- |

where $\Delta_{nonsyn,del}$ is the total change in deleterious nonsynonymous mutations at each timestep due to either substitution or backmutation. The difference between Equation 5 and Equation S2 is the inclusion of a deleterious nonsynonymous mutation term that decreases shedding duration.

For the three gateway mutations, we assume that the selection coefficient is proportional to the relative rate of fixation compared to A481G, the fastest gateway mutation to fix (average 7 days). This allows us to redefine their selection coefficients using a single selection coefficient ($s_{dur, point}$):

| $s_{inf, gateway}=s_{dur,point}*\frac{\log\left( \lambda_{gateway} \right)}{\log\left( \lambda_{A481G} \right)}$ | (S3) |
| --- | --- |

Note that $s_{dur,point}$ is equivalent to $s_{dur,A481G}$. Relating $s_{gateway}$ to the relative ratio of log fixation rates was decided empirically and resulted in better calibration fits. We calibrated simulated shedding durations using four parameters: $s_{dur,point}$, $s_{dur,nonsyn_{del}}$, $\mu_{S2}$ (the base shedding duration of unevolved Sabin 2, $G_{0,0,0,0,0,0}$) and $\sigma$ the standard deviation in shedding duration. To calibrate these three parameters, we utilized two sets of simulations: one where we replicate the shedding duration of Sabin 2 vaccinees and one where we replicate the shedding duration of WPV infected individuals. Each simulation simulates 1000 infections and allows the shedding duration to be affected by gateway mutation reversion and deleterious nonsynonymous mutation accumulation.

We simulated 1000 Sabin 2 vaccinations in immunologically naïve individuals (Nab = 1) and allowed newly acquired mutations to extend the shedding duration as defined by **Equation S2**. Once all vaccinated individuals stop shedding, we compared simulated shedding durations against 1000 samples from the lognormal distribution describing the shedding durations following clinical Sabin 2 vaccination (median = 30.3, std = 1.86) (Famulare et al. 2018). We also simulated WPV shedding durations by infecting 1000 immunologically naïve individuals with $G_{1,1,1,9. X,X}$. This genome represents the average, phenotypically relevant genotype of fully reverted cVDPV2 sequences. Note that we assume nine deleterious nonsynonymous mutations because it represents the average steady-state value in our simulated genomes after all intra-host substitution rates were calibrated. The number of neutral nonsynonymous mutations and synonymous are denoted by X and assigned an arbitrary value during simulations because they do not affect shedding duration. These shedding durations were compared against 1000 samples from the lognormal distribution describing shedding duration in natural WPV infections (median = 43.0, std = 1.69) (Famulare et al. 2018).

Posteriors for $\mu$, $s_{dur,point}$,and $s_{dur,nonsyn_{del}}$ were generated using ABC-SMC with 300 particles and three iterations. Priors were obtained by running a simplified version of the simulations described above, where we 1) assume mutations always arise at their average fixation times, 2) assume that WPV does not acquire additional nonsynonymous mutations (ie $G_{1,1,1,9. X,X}$remains constant), 3) obtain parameter estimates by minimizing the least-squares difference between simulated and observed means. For the ABC-SMC, distance was defined using a pseudo-log likelihood function:

| $L= -\frac{n}{2}\ln\left( 2\pi\right)-\frac{n}{2}\ln\left( \hat{v} \right)-\frac{1}{2\sigma^{2}}\sum_{j-1}^{n} \left( x_{j}-\hat{m} \right)^{2}$ | (S4) |
| --- | --- |

where $x_{j}$ denotes a random sampling form the target lognormal distribution, n is the total number of samples made, $\hat{m}$ is the mean and $\hat{v}$ is the variance of the simulated shedding durations. Error thresholds for each iteration were empirically generated by drawing 1000 parameter sets from our priors and set at the 30^th^, 10^th^, and 5^th^ percentiles.

Our point estimate for $s_{nonsyn_{del}}$ was -0.006 (-0.04, 0.03) but is too small to have a noticeable effect on shedding duration (**Supplemental Figure 7**). Overall, there was insufficient evidence to definitively identify $s_{nonsyn_{del}}$ as increasing or decreasing shedding duration, as the posterior occupies both positive and negative values. As a result, we simplified our final shedding duration model and assert $s_{nonsyn_{del}}$ as zero (equivalent to **Equation 5**). Final calibration estimates are in **Supplemental Table 3**.

*Identifying the impact of immunity on shedding duration*

Thus far, our calibration has focused on the shedding duration in immunologically naïve individuals. To calibrate shedding duration in individuals, we also needed to re-evaluate $\delta$, the parameter that governs the decrease in shedding duration due to immunity (**Equation X**). To calibrate $\delta$, we simulated the expected shedding durations for individuals with $N_{ab}$ ranging from 1-4096. Using all previously calibrated parameter estimates, we used ABC-SMC with 300 particles and three iterations to compare simulated shedding durations with the expected shedding durations for Sabin 2 reported in (Famulare et al. 2018) for each $N_{ab}$ titer examined. A prior was obtained by performing a parameter sweep across $\delta$, ranging from 0-2; our previous estimate for $\delta$ was 1.16 (Famulare et al. 2018). Distance was calculated as the least square difference in average shedding duration. Error thresholds for each iteration were empirically generated by drawing 1000 parameter sets from our priors and set at the 30^th^, 10^th^, and 5^th^ percentiles. Priors and the final posterior estimates are reported in **Supplemental Table 4**.

*Genotype to Phenotype: Calibrating Infectiousness*

WPV is approximately four times more infectious than Sabin 2 (2.3 CCID50 units for WPV and 8 CCID50 units for Sabin 2). As with shedding duration, we attribute this increase in infectiousness (decrease in CCID50 units) to the acquisition of the three gateway mutations and the accumulation of deleterious nonsynonymous mutations. This is governed by $\beta_{g}$ (**Equation 8**), which describes the change in infectiousness associated with each mutation. Our goal was to infer $s_{inf,A481G}$, $s_{inf,U2909C}$, $s_{inf,U398C}$, and $s_{inf, nonsyn_{del}}$.

Like with shedding duration, we simplify parameter calibration by assuming the selection coefficients for each of the gateway mutations is proportional to their relative fixation rates:

| $s_{inf, gateway}=s_{inf,point}*\frac{\lambda_{gateway}}{\lambda_{A481G}}$ | (S5) |
| --- | --- |

Note that the fixation rate ratios are not in log space. Infectiousness is determined only at the onset of infection. Point estimates were obtained by minimizing **Equation S6** using a numerical approximation method (scipy.optimize.minimize, method = ‘TNC’):

| $\beta_{wpv}=\frac{\beta}{\left( 1+s_{inf,A481G} \right)^{1} \left( 1+s_{inf,U2909C} \right)^{1} \left( 1+s_{inf,U398C} \right)^{1} \left( 1+s_{inf,nonsyn_{del}} \right)^{9}}$ | (S6) |
| --- | --- |

where $\beta$ and $\beta_{wpv}$ are substituted with the average estimates obtained from various OPV trials (Famulare et al. 2018). Note that the WPV genotype is again defined as $G_{1,1,1,9. X,X}$. Point estimates are provided in **Supplemental Table 5**.

*Calibration to mOPV2 clinical trial data*

We previously developed a multiscale, household demographic transmission model (Famulare et al. 2021) that simulates changing poliovirus immunity and transmission with different contact rates across different demographic scales: 1) household, 2) bari, an intergenerational living arrangement unique to rural Bangladesh, 3) village, and 4) region. Contact rates and fecal-oral doses were calibrated to Sabin 2 shedding following a monovalent, Sabin 2 Oral Poliovirus (mOPV2) campaign performed in Matlab, Bangladesh. After incorporating Sabin 2 evolution, we recalibrated our multiscale transmission model by performing a parameter sweep across the fecal-oral dose parameter. All other parameters regarding demographics and contact rates were not recalibrated. Our estimate for the fecal oral dose was 4.0-7g/contact (2.8e-7, 6.1e-7) (**Supplemental Figure 8**), which is about an order of magnitude lower than our previous estimate (1.25e-6 g/contact).

**Supplemental Works Cited**

Famulare M, Chang S, Iber J, Zhao K, Adeniji JA, Bukbuk D, Baba M, Behrend M, Burns CC, Oberste MS. 2015. Sabin Vaccine Reversion in the Field: a Comprehensive Analysis of Sabin-Like Poliovirus Isolates in Nigeria. *J Virol* [Internet] 90:317–331. Available from: https://www.ncbi.nlm.nih.gov/pubmed/26468545

Famulare M, Selinger C, McCarthy KA, Eckhoff PA, Chabot-Couture G. 2018. Assessing the stability of polio eradication after the withdrawal of oral polio vaccine. *PLOS Biology* [Internet] 16:e2002468. Available from: https://doi.org/10.1371/journal.pbio.2002468

Famulare M, Wong W, Haque R, Platts-Mills JA, Saha P, Aziz AB, Ahmed T, Islam MO, Uddin MJ, Bandyopadhyay AS, et al. 2021. Multiscale model for forecasting Sabin 2 vaccine virus household and community transmission. *PLOS Computational Biology* [Internet] 17:e1009690. Available from: https://journals.plos.org/ploscompbiol/article?id=10.1371/journal.pcbi.1009690

Jorba J, Campagnoli R, De L, Kew O. 2008. Calibration of Multiple Poliovirus Molecular Clocks Covering an Extended Evolutionary Range. *Journal of Virology* 82:4429 LP – 4440.

Sadegh M, Vrugt JA. 2014. Approximate Bayesian Computation using Markov Chain Monte Carlo simulation: DREAM(ABC). *Water Resources Research* 50:6767–6787.

Wassilak S, Pate MA, Wannemuehler K, Jenks J, Burns C, Chenoweth P, Abanida EA, Adu F, Baba M, Gasasira A, et al. 2011. Outbreak of type 2 vaccine-derived poliovirus in Nigeria: emergence and widespread circulation in an underimmunized population. *J Infect Dis* 203:898–909.
