# Supplementary figures and images for: From vaccine to pathogen: Modeling Sabin 2 vaccine virus reversion and evolutionary epidemiology in Matlab, Bangladesh
Source: Virus Evol. 2023 Jul 8;9(2):vead044. doi: 10.1093/ve/vead044 (PMC10491863; doi:10.1093/ve/vead044)

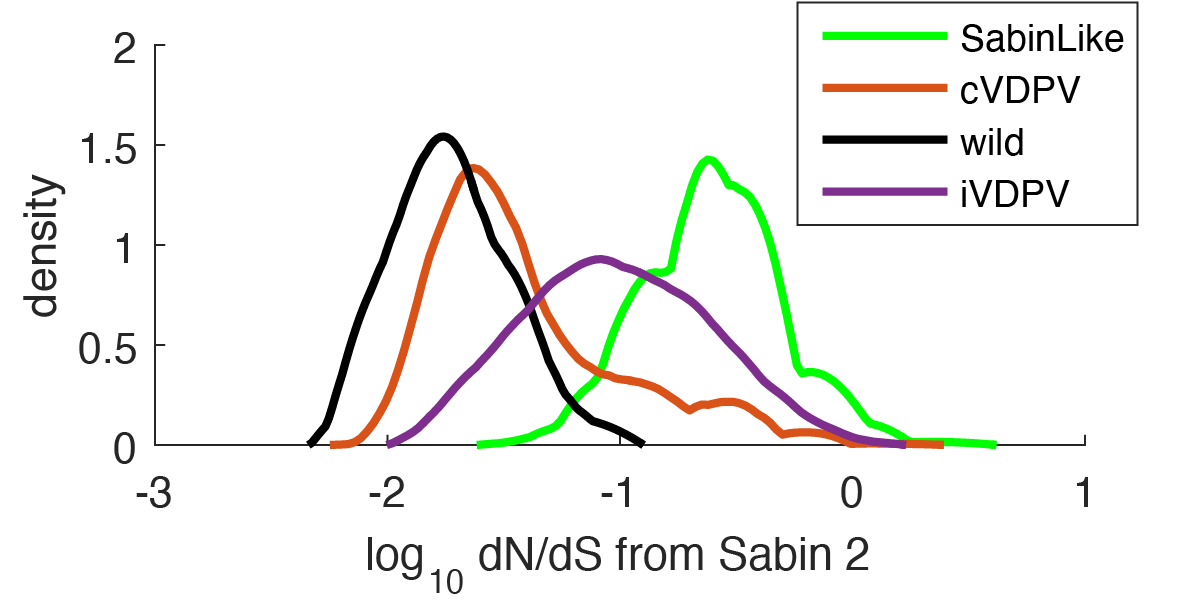

Supplement: vead044_Supp [file vead044_supp.zip › Supplemental Figure 1 DnDs.tif]

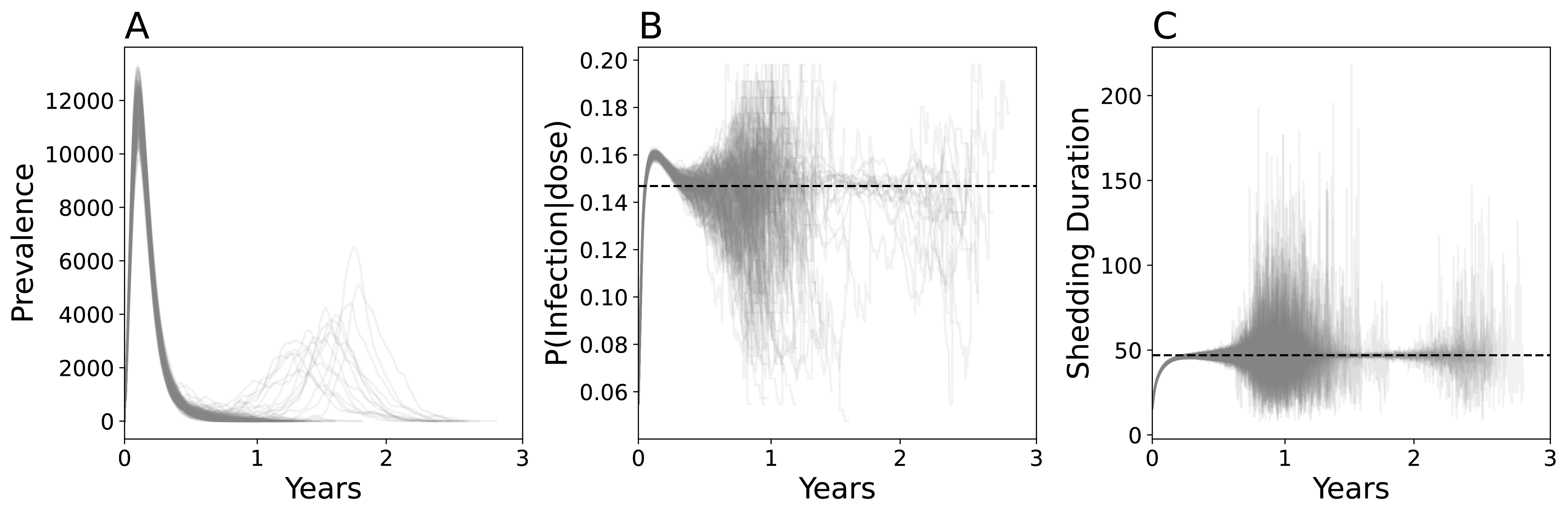

Supplement: vead044_Supp [file vead044_supp.zip › Supplemental Figure 2 variance traces.tif]

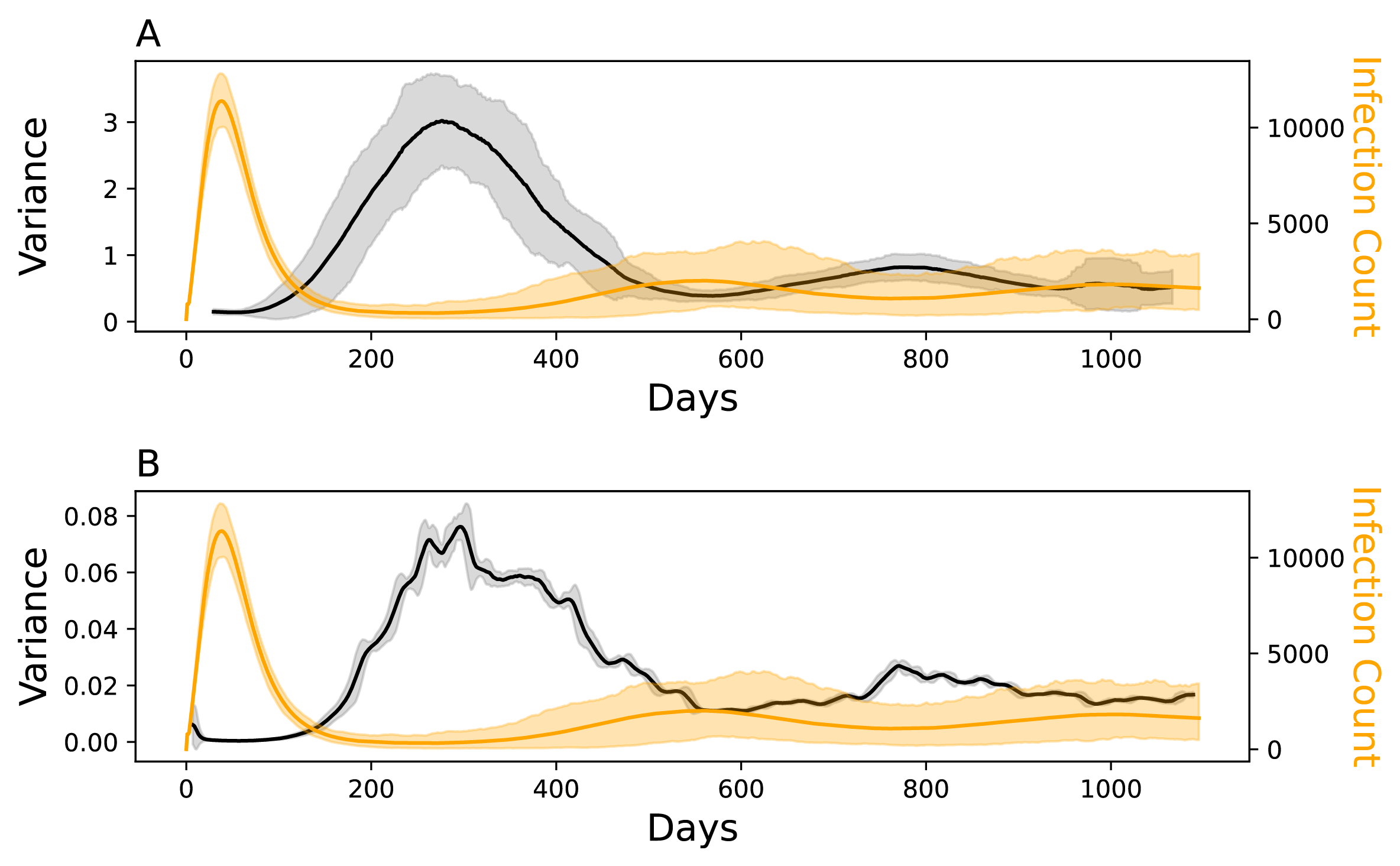

Supplement: vead044_Supp [file vead044_supp.zip › Supplemental Figure 3 variance condition.tif]

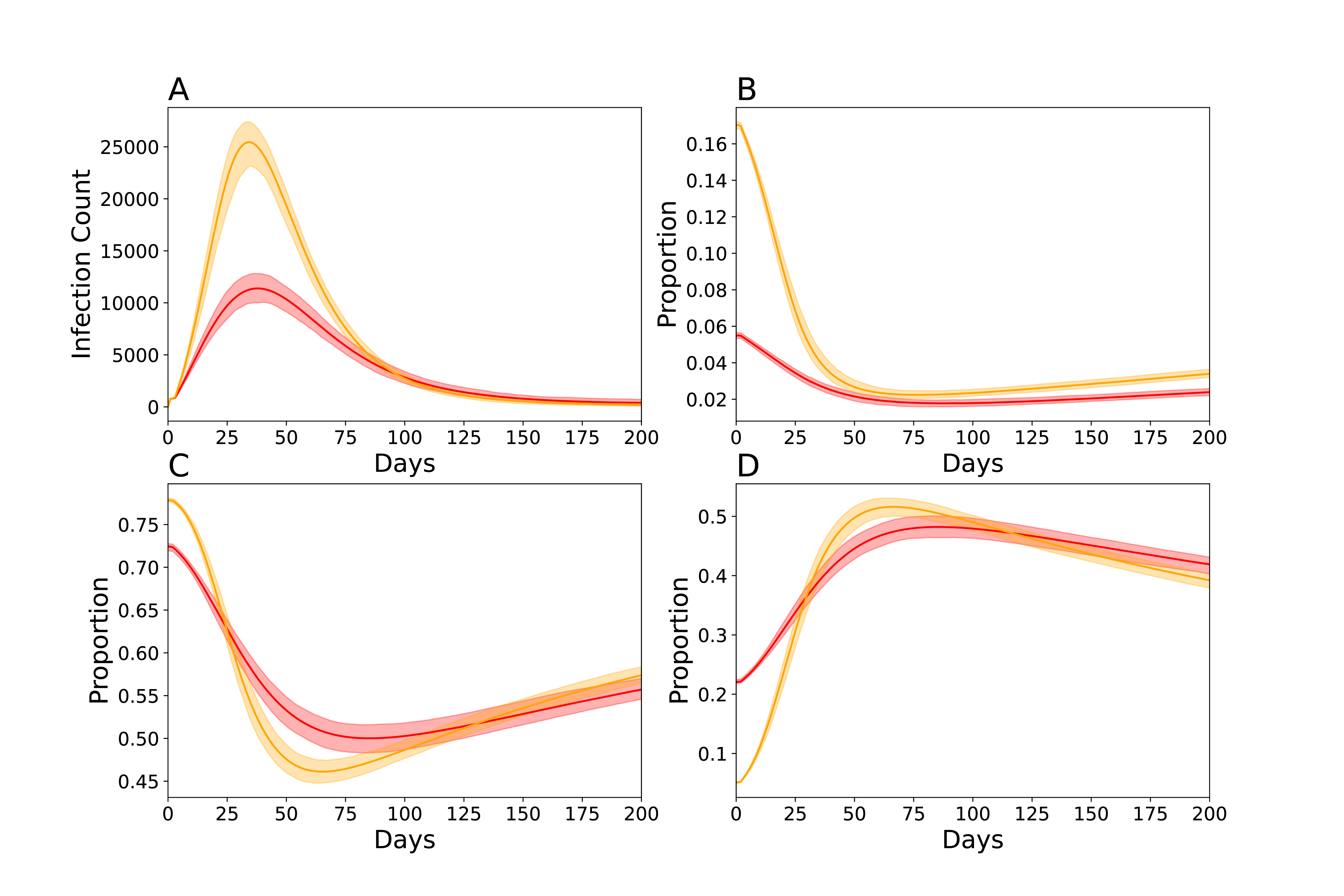

Supplement: vead044_Supp [file vead044_supp.zip › Supplemental Figure 4.tif]

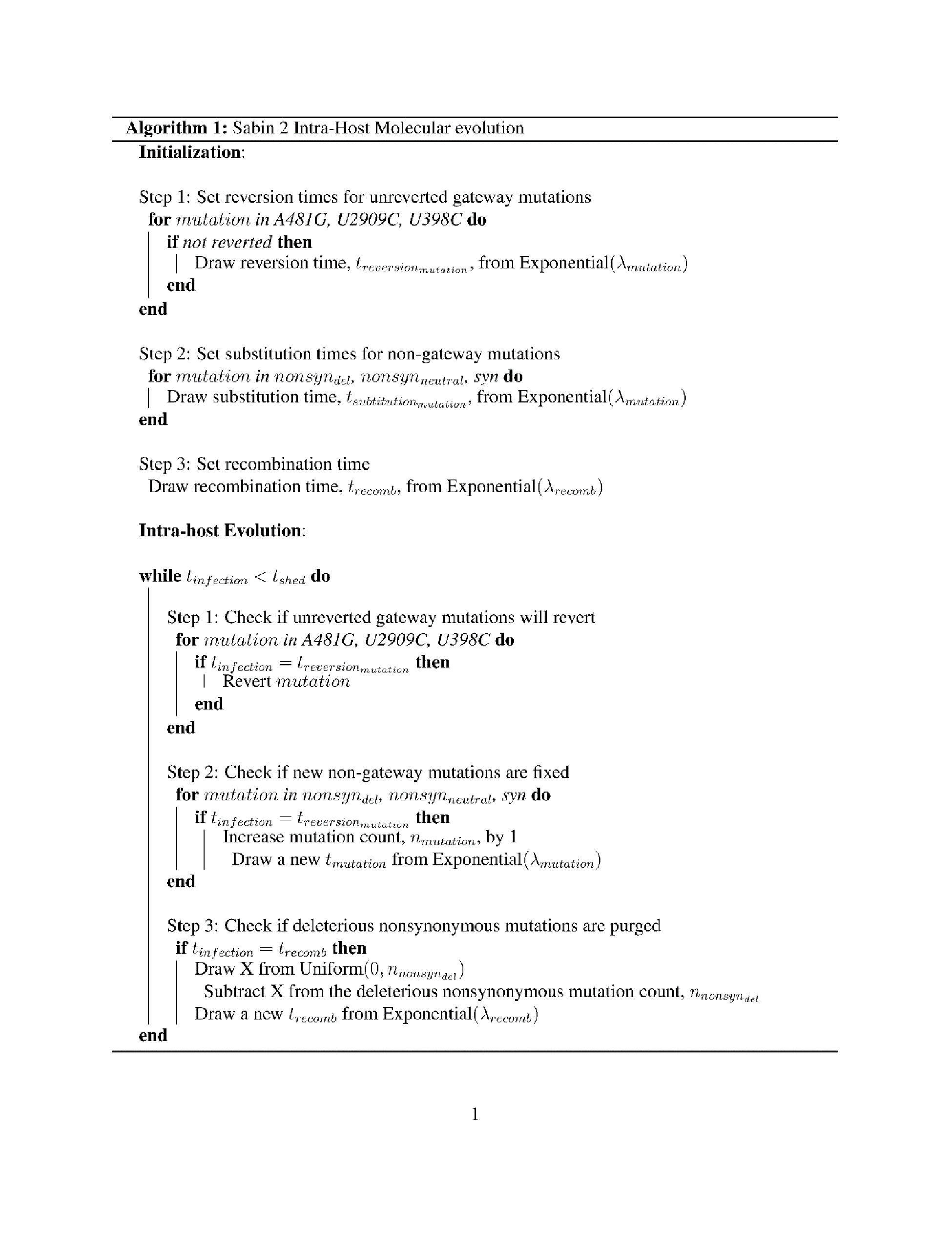

Supplement: vead044_Supp [file vead044_supp.zip › Supplemental Figure 5.tif]

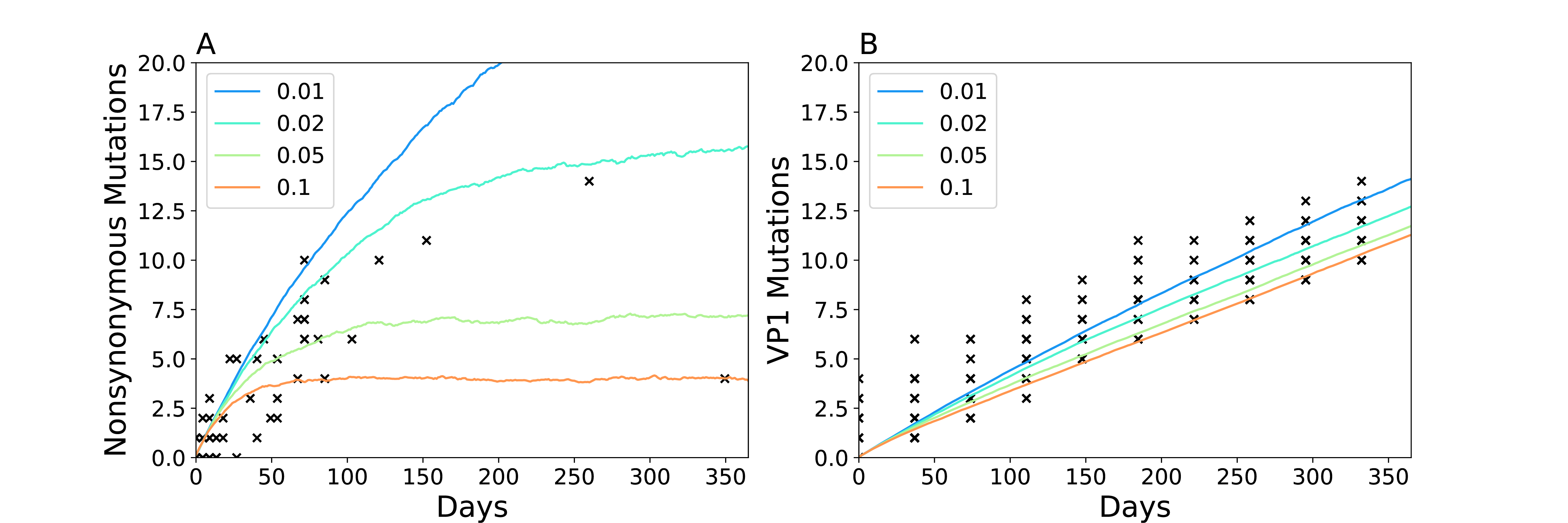

Supplement: vead044_Supp [file vead044_supp.zip › Supplemental Figure 6 parameter sweep.tif]

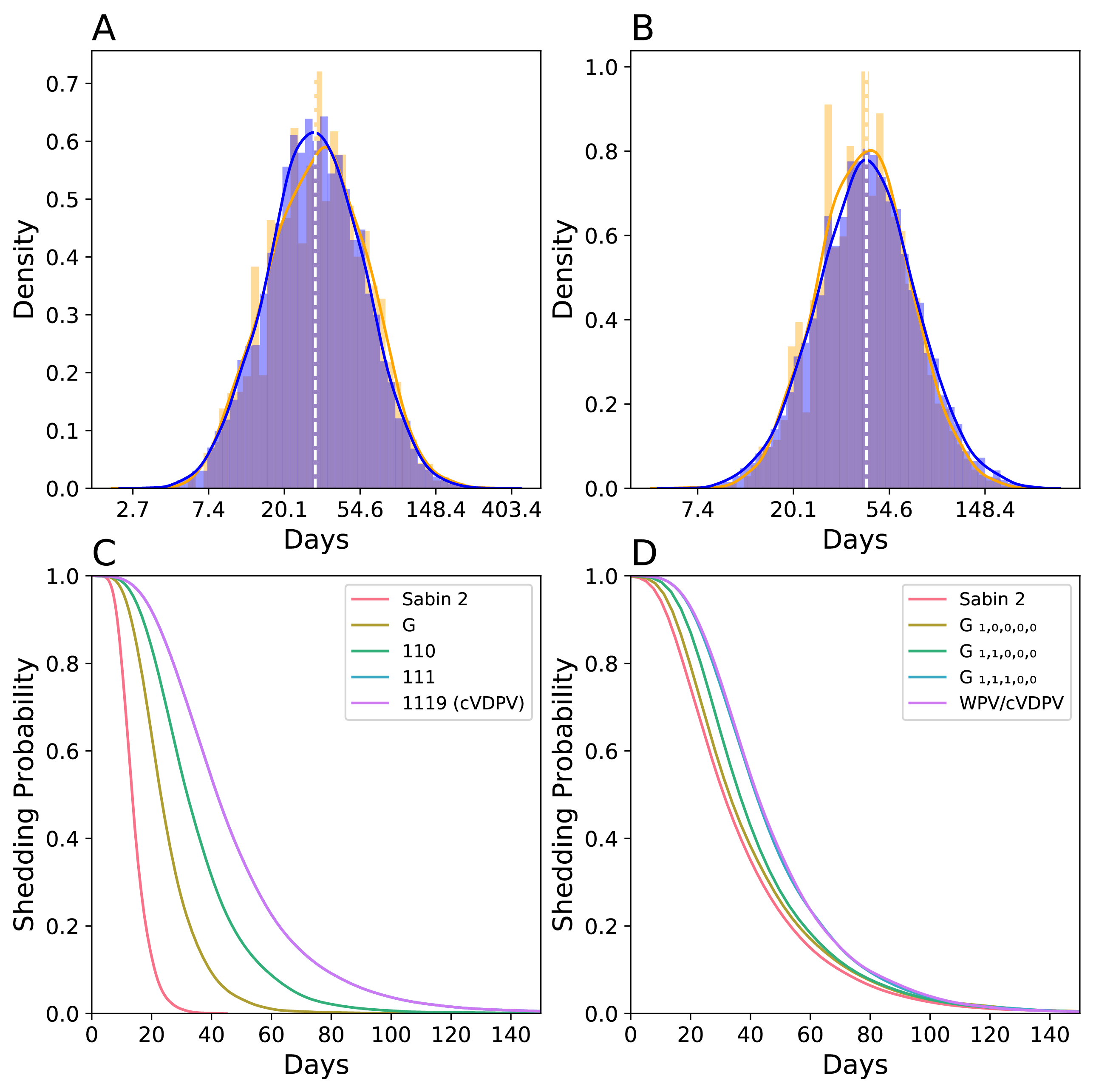

Supplement: vead044_Supp [file vead044_supp.zip › Supplemental Figure 7 shedding.tif]

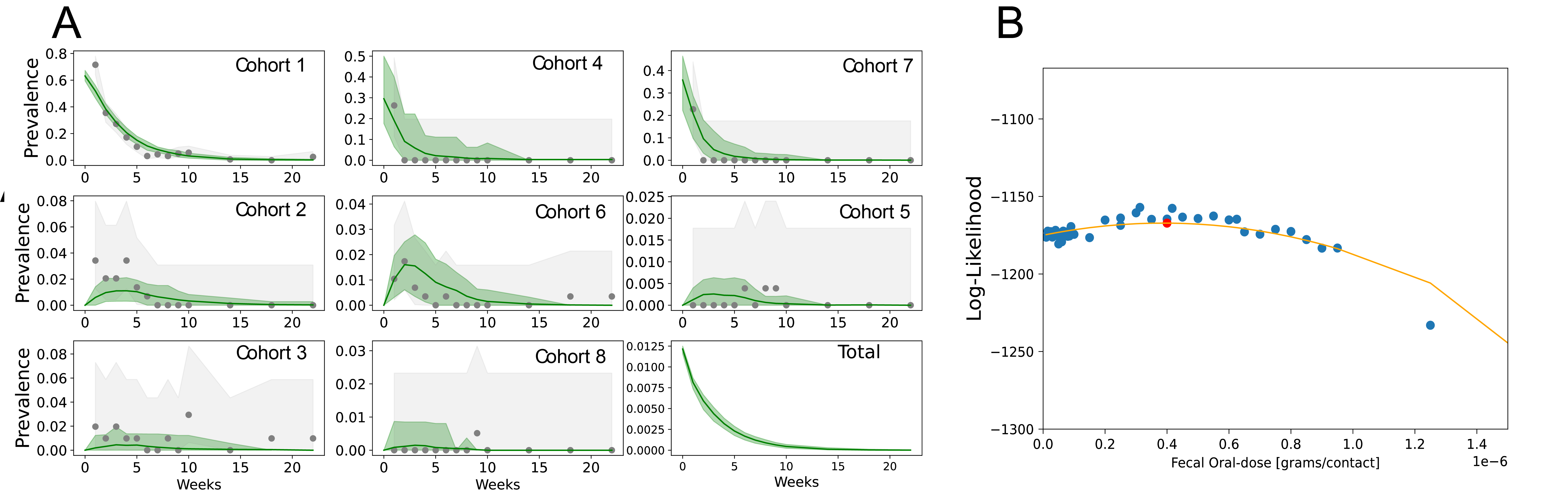

Supplement: vead044_Supp [file vead044_supp.zip › Supplemental Figure 8 Fecal_calibration.tif]
